# Distinguishing near- versus off-critical phase behaviors of intrinsically disordered proteins

**DOI:** 10.1088/1361-6633/ae70d6

**Published:** 2026-06-08

**Authors:** Gaurav Mitra, Souradeep Ghosh, Kiersten M Ruff, Ruoyao Zhang, Gaurav Chauhan, Rohit V Pappu

**Affiliations:** 1 Department of Biomedical Engineering and Center for Biomolecular Condensates, Washington University in St. Louis, St. Louis, MO, United States of America; 2 Department of Chemical Engineering, Indian Institute of Technology, Indore, India

**Keywords:** critical points, universality, intrinsically disordered proteins, phase separation, percolation

## Abstract

Intrinsically disordered prion-like low complexity domains (PLCDs) drive phase transitions that underlie the biogenesis of many biomolecular condensates. Here, we report results from large-scale Monte Carlo simulations on lattices aided by computations of Binder cumulants and rigorous finite-size scaling. These approaches enable accurate mapping of the critical regime and computations of the full binodal of an archetypal PLCD. This weakly associating polymer undergoes phase separation coupled to percolation. Between the lowest temperature and the critical point, the concentrations along the left arm of the binodal vary by four orders of magnitude. The overlap line intersects the left arm of the binodal well below the critical point. This, taken together with the intersection of the percolation line and the left arm of the binodal, leads to demarcation of the binodal into three regimes. Regime I is farthest from the critical point. Here, the coexisting dilute phase is akin to a gas of dispersed polymers. The dilute arm of the binodal lies above the overlap line in Regimes II and III. Here, the semidilute nature of dilute phases enables clustering of polymers that is enhanced by intermolecular associations. The coexisting dense phases form confined percolated networks in Regimes I and II. In Regime III, which is closest to the critical point, the dense phase becomes unconfined and fragmented, and the system is defined by two interconnected, system-spanning networks. In addition to mapping the critical point accurately, we evaluated methods for identifying the theta temperature. We find that scaling approaches based on assumptions from two-parameter theories for homopolymers yield erroneous estimates of the theta temperature of an archetypal PLCD. Accurate estimation of the theta temperature requires direct calculation of the temperature dependence of the two-body interaction coefficient. We discuss implications for inferring solvent quality from scaling analysis of segmental distances of disordered proteins.

## 1. Introduction

Biochemical reactions in living cells are organized in space and time by the reversible formation of membraneless bodies known as biomolecular condensates [1, 2]. Phase transitions drive condensate formation [3–5] and multivalence is a defining hallmark of proteins that drive condensation [1, 5, 6]. For proteins, multivalence comes in different flavors [7] and this includes: multiple protein-protein interaction domains tethered by intrinsically disordered linkers [5, 8–10], oligomeric proteins comprising folded domains with or without intrinsically disordered regions [11–15], or intrinsically disordered proteins (IDPs) featuring multiple cohesive motifs that provide specificity of associations [16–20].

Phase separation coupled to percolation (PSCP) is the process that explains the complete set of known phase transitions that underlie condensate formation [5, 6, 8, 9, 21–25]. In this process, multivalence of specific motifs or domains enables reversible associations that lead to the formation of clusters of different sizes in solution [26]. As concentrations increase above a threshold known as the gel point or bond percolation threshold ($c_\mathrm{p}$) [21, 27], there is a finite probability of forming clusters that can grow to become system-spanning. However, at temperatures below the critical temperature $T_\mathrm{c}$, the growth of clusters in solution is typically suppressed because the system crosses the solubility limit [26, 28–31]. Hence, the overall free energy of the system is minimized by separation of the entire solution into coexisting dense and dilute phases via a process known as phase separation [6, 32]. For systems where the associations are homotypic, the threshold concentration for phase separation is the saturation concentration or $c_\mathrm{sat}$. If a blend of homotypic and heterotypic associations is involved [33], then the solubility limit is defined by the solubility product [34–36], with different components contributing differently [36, 37]. For condensation via PSCP, the macromolecular concentration within dense phases will typically be above $c_\mathrm{p}$, and the dense phase will be a percolated network that resembles a confined physical gel [9, 30, 38]. Whether the confined physical gel is a weak or strong gel will depend primarily on the strengths of the physical crosslinks between cohesive motifs. It will also depend on the polymer concentration within the dense phase and the balance of chain–chain and chain–solvent interactions [39].

**Figure 1.**
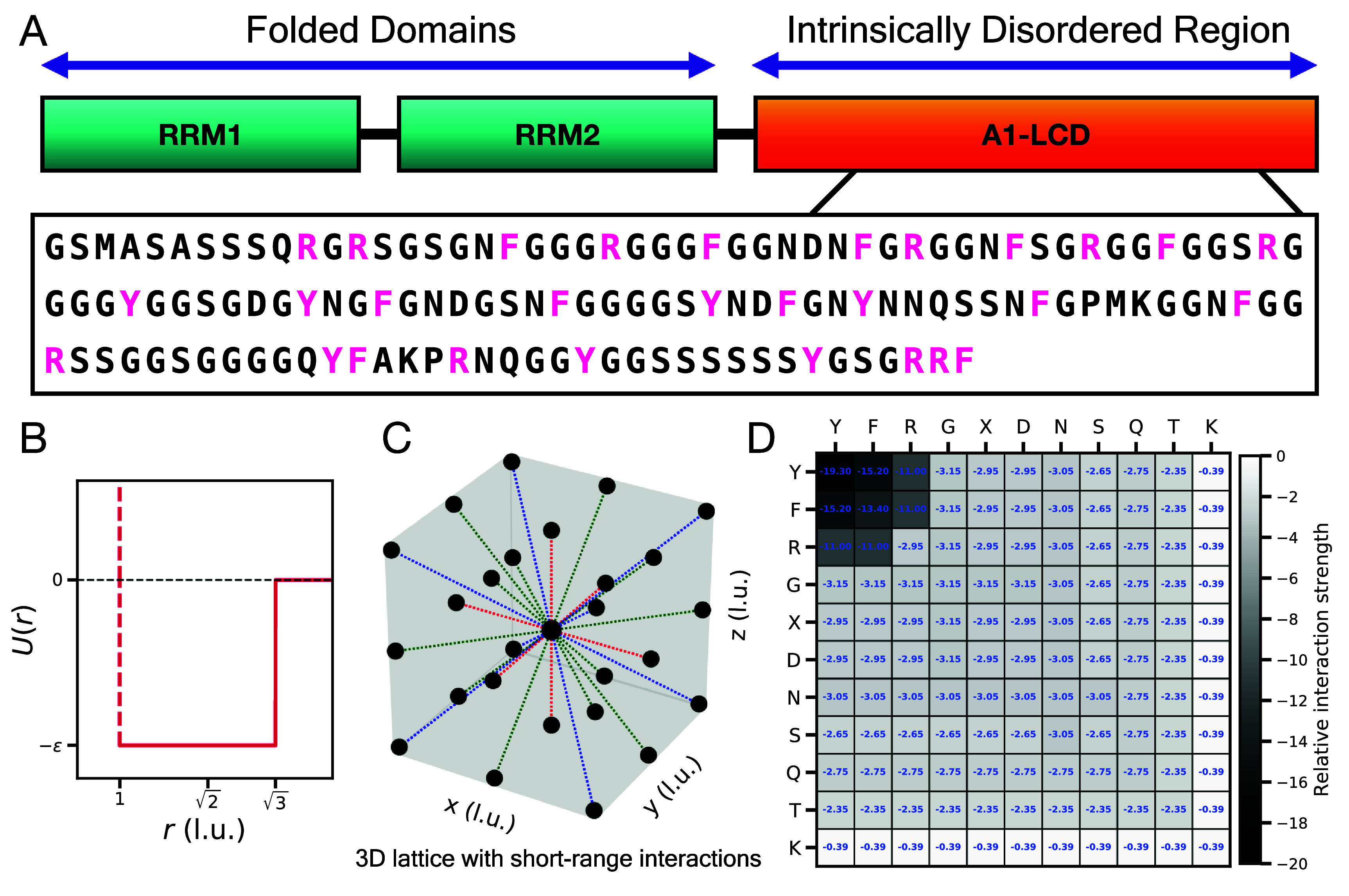
Details of the coarse-grained lattice-based Monte-Carlo simulations. (A) Architecture of the hnRNP-A1 protein and amino acid sequence of A1-LCD. hnRNP-A1 comprises the folded RNA recognition motifs (RRMs) and the PLCD (prion-like low complexity domain), which is designated as A1-LCD. The amino acid sequence of A1-LCD, which has 137 residues, is also shown in the box. Sticker residues in the A1-LCD sequence were identified in previous studies [51, 52], and these are tyrosine (Y), phenylalanine (F), and arginine (R), which are colored in magenta to delineate them from the rest of the residues (colored in black). (B) Pairwise energy of interaction shown as a function of the distance between any two beads on the lattice (See Methods section). (C) Schematic of a cubic lattice with short-range interactions where each bead has 26 possible neighbors at distances of $1$ l.u. (colored by red dotted lines), $\sqrt{2}$ l.u. (colored by green dotted lines), or $\sqrt{3}$ l.u. respectively (colored by blue dotted lines). (D) Matrix showing the pairwise interaction strengths used in the LaSSI computational model. ‘X’ is used to indicate any amino acid for which a specific interaction is not defined. The values for the relative interaction strengths for each residue pair are shown inside the squares of the matrix grid. The energies are unitless. Moves are accepted or rejected by referencing to thermal energy $k_\mathrm{B}T_\mathrm{s}$, where $k_\mathrm{B} = 1$ and $T_\mathrm{s}$ is the simulation temperature.

It has been proposed that condensates in living cells have the potential to be in the vicinity of thermodynamic critical points [40–47]. Being close to the critical point would imply that condensates behave like systems that are on the edge of stability, characterized by large fluctuations of the order parameter. For condensates, the relevant order parameters are two-fold: they are the density differences of the condensate-driving macromolecules and solution components across coexisting phases [32], and the degree of clustering and networking of molecules [27]. Importantly, the one-phase regimes for temperatures near the critical point versus far away from the critical point are expected to be very different [43, 48]. Thus, the properties of the coexisting dilute phases are likely to be useful and measurable signatures of near- versus off-critical behaviors of condensates. To set up expectations for a one-component system, we turned to simulations and mapped the complete phase behavior of A1-LCD, which is an archetypal IDP. This is the intrinsically disordered prion-like low complexity domain (PLCD) from the protein hnRNP-A1 (figure 1(A)) [49]. A1-LCD and other PLCDs are exemplars of *weakly associating polymers* [22]. An associating polymer is defined by two distinct energy scales namely, specific and stronger sticker-sticker interactions versus non-specific and typically weaker spacer-mediated interactions that determine the two- and three-body interaction coefficients [26]. In weakly associating polymers, the strengths of sticker-sticker interactions are in the range of 3–5  $k_\mathrm{B}T$ [26]. In contrast, for strongly associating polymers such as ionomers, the sticker-sticker interaction strengths are $ > 10\;k_\mathrm{B}T$ [26, 30, 50]. Through systematic mutagenesis experiments, the primary stickers in A1-LCD were found to be the aromatic residues (Phenylalanine and Tyrosine) whereas Arginine (Arg) residues serve as auxiliary stickers in these PLCDs [17, 51, 52]. The identities of stickers and spacers are not immutable. Instead, they are context-dependent [6]. For example, in the context of PLCDs, the aromatic residues may be viewed as the primary stickers and Arg residues as auxiliary stickers [51, 52]. Similar results were found for the disordered region of synapsin [53]. Conversely, in full-length fused in sarcoma (FUS), the primary stickers were found to be Arg and Tyr residues [16]. The identities of stickers versus spacers can be predicted using methods that identify non-random features, known as molecular grammars, within disordered regions of proteins [54–56].

Coarse-grained simulations have been used to map binodals for an assortment of systems [17, 20, 46, 52, 57–62]. Here, we assess the mapping of critical points using approaches established in the literature [63], and then examine the accuracy of this method by performing large-scale simulations. Rigorous finite size scaling based on analysis of Binder cumulants [64] yields accurate estimates of the critical point. We use this approach to map the full binodal of A1-LCD and demonstrate how the percolation and overlap lines intersect the dilute arm of the coexistence curve (binodal). We identify three distinct regimes, which are characterized by distinct, regime-specific cluster-size distributions in the coexisting dilute phases. We also estimate the theta temperature as the point where the two-body interaction coefficient ($B_2$) for a pair of chains equals zero. We find that the temperature where this criterion is met is very different from the apparent theta temperature inferred by analysis of the scaling of mean segmental distances $R(s)$ as a function of $s$, the length of a segment.

## 2. Methods

**Figure 2.**
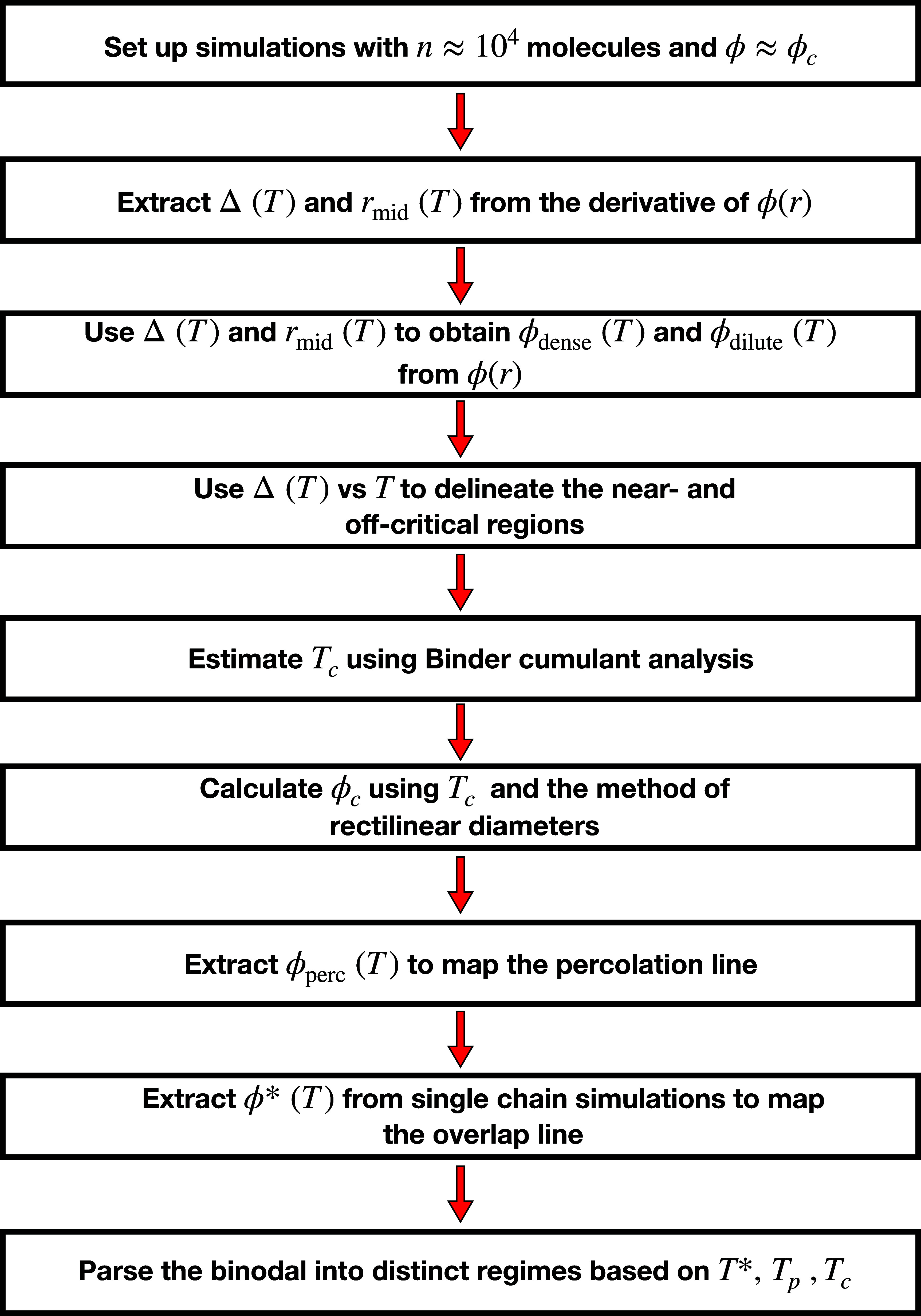
Flowchart showing the nine-step simulation and analysis protocol to map the full binodal for WT A1-LCD. Details of the protocol are provided in the methods section of the main text.

Our coarse-grained lattice-based Monte Carlo (MC) simulations [65–67] employ the LaSSI simulation engine [27, 52], which is based on a generalization of the bond fluctuation model [68, 69]. A single A1-LCD molecule comprises 137 beads, one for each of the residues, and each residue is a single bead on a lattice. For the inter-residue potentials (figures 1(B) and (D)), we used a short-range potential function that was parameterized using a Gaussian process Bayesian optimization procedure [52, 70]. The parameters are transferable unto other PLCDs but not to other systems [52, 62]. To map the critical region, we used finite-size scaling that relies on the quantification of density fluctuations via analysis of Binder cumulants [64, 71]. We used a nine-step protocol, which is followed sequentially to arrive at a complete characterization of the binodal of A1-LCD (figure 2). The key considerations are as follows: Obtaining converged estimates of mean densities, density fluctuations, and higher-order moments, which are imperative for mapping critical regions and percolation lines, requires that the simulation cells encompass at least ${\sim}6 \times 10^3$ A1-LCD molecules. The box sizes need to be large because of the high volume fractions near critical points, and the boxes need to be cubic with periodic boundaries to avoid confinement or symmetry-breaking artifacts. As a result, the number of MC steps required to obtain converged estimates is $O(10^{11})$. The simulation system includes $10^4$ A1-LCD molecules for each simulation temperature $T_\mathrm{s}$. For every value of $T_\mathrm{s}$, we performed at least three independent MC simulations, each initiated with different random seeds. Each simulation comprises $6 \times 10^{11}$ MC steps. In the currency of MD simulations, $6 \times 10^{11}$ MC steps would translate to simulation times of ${\sim} 3$ ms assuming an integration time step of 5 fs. Additional simulations of equivalent length (for more temperatures in the critical region) were performed when the Binder cumulant analysis needed to be deployed. For computing binodals as a function of temperature, we used cubic boxes that were 240 lattice units ($\mathrm{l.u.}$) to a side. In Å, this would translate to ${{\sim}} 960$ Å. For the finite-size scaling analysis, we quantified Binder cumulants using sub-boxes whose dimensions span the range of $L \approx 50 - 200\;\mathrm{l.u.}$

### 2.1. LaSSI simulations

The lattice-based MC simulations were performed following the approach of Farag *et al* [52, 62]. Beads occupy positions on a cubic lattice, and the energy of interaction is $-\varepsilon_{ij}$ (figures 1(B) and (C)) for beads of type $i$ and $j$ if the distance between the beads on the lattice is 1, $\sqrt{2}$, or $\sqrt{3} \; \mathrm{l.u.}$ apart. Otherwise, the attractive pairwise interaction energies are zero. The values of $-\varepsilon_{ij}$ were those of Farag *et al* [52] (figure 1(D)). The energies were parameterized to reproduce the sequence specificity of measured radii of gyration [51] and the parameterization scheme was based on the Gaussian process Bayesian optimization approach of Ruff *et al* [70]. We analyzed the results from two sets of simulations, one that includes 200 A1-LCD molecules in a cubic box ($L_\mathrm{sim} = 120\;\mathrm{l.u.}$) and the other that includes $10^4$ molecules ($L_\mathrm{sim} = 240\;\mathrm{l.u.}$).

### 2.2. Conversion from simulation to experimental units

Scaling factors are required to convert the computed coexistence curves from simulation temperatures into degree Kelvin and volume fractions into molar units. For the latter, we followed Wei *et al* [40] and Farag *et al* [52]. A volume fraction of 1.0 corresponds to a mass concentration of 1310 mg ml$ ^{-1}$. Following Farag *et al* [52], we used a multiplicative factor of 5.6 to convert the simulation temperature in LaSSI (which is written in units of $k_\mathrm{B} = 1$) to degrees Kelvin. Farag *et al* derived this factor as follows: Binodals extracted from LaSSI simulations were converted into mass concentrations along the abscissa, and the ordinate was multiplied by a conversion factor that maximized the overlap with the measured binodals for A1-LCD [17, 52]. This conversion, which was derived for A1-LCD, was then applied without further modification to more than 30 different sequences derived from the A1-LCD parent [51, 52] and to the LCD of FUS [62]. A fixed density scaling factor (0.6) was used to convert volume fraction from lattice units to volume fraction as measured experimentally or, equivalently, from simulation concentrations in (M) to experimental concentrations measured in (M) [17, 52]. Lengths in lattice units were converted to real units using a lattice spacing of 4 $\mathrm{\unicode{x00C5}}$ per lattice unit. This spacing was chosen because it corresponds to the average distance between adjacent $\mathrm{C}_{\alpha}$ atoms ($3.8-4.0 \;\mathrm{\unicode{x00C5}}$) [72, 73]. Consequently, one lattice site represents a single residue both in volume and in bond length, yielding physically realistic chain dimensions. Note that in all our calculations, we quantify the densities of polymer beads and not of molecules. Together, these conversions enable direct comparison between lattice simulations and experimental data. LaSSI simulations to study the phase behaviors were performed for $46 \unicode{x2A7D} T_\mathrm{s} \unicode{x2A7D} 64$ ($257.60\;\mathrm{K} \unicode{x2A7D} T \unicode{x2A7D} 358.40\;\mathrm{K}$, where $T$ is the temperature in Kelvin).

### 2.3. Protocol for accurately mapping the critical regime and estimating critical parameters

**Step 1** involves setting up simulations of the appropriate size (box size and numbers of molecules) and length (number of MC steps). **Step 2** is the extraction of the interfacial width $\Delta(T)$ and the midpoint of the interface $r_\mathrm{mid}$ from the radial density profiles. **Step 3** uses the $T$-dependent radial density profiles and the estimated $T$-dependent values of $\Delta(T)$ and $r_\mathrm{mid}$ to extract $\phi_\mathrm{dense}(T)$ and $\phi_\mathrm{dilute}(T)$ using the Fisk–Widom function [74]. **Step 4** delineates the critical and off-critical regimes by analyzing the variation of $\Delta(T)$ with $T$, quantifying $T_{\mathrm{crossover},\Delta}$, and designating the region above $T_{\mathrm{crossover},\Delta}$ as the critical region. **Step 5** deploys the analysis of Binder cumulants, which requires considerable additional sampling for temperatures closer to the critical region, to estimate $T_\mathrm{c}$. This knowledge can be used to estimate $\phi_\mathrm{c}$ directly using the full binodal. As an additional route to estimating $\phi_\mathrm{c}$, **Step 6** uses the known $T_\mathrm{c}$ to calculate $\phi_\mathrm{c}$ via the method of rectilinear diameters. **Step 7** deploys sampling at various densities to extract the temperature-dependent percolation thresholds, the locus of which constitutes the percolation line. **Step 8** estimates the overlap line by performing single-chain simulations at different temperatures to estimate $\phi^\ast(T)$. **Step 9** quantifies the percolation and overlap lines, and estimates the points where these lines intersect the binodal. All steps of this protocol are shown in the flowchart in figure 2 and details of each step may be found in the *SI appendix*.

## 3. Results

### 3.1. System size considerations for LaSSI simulations

To discern the optimal box size and numbers of A1-LCD molecules ($n$) that are needed to map critical regions, we first estimated the critical volume fraction ($\phi_\mathrm{c}$) by fitting the computed binodal from simulations with 200 molecules. The fitting was performed using a modified Flory–Huggins model (see *SI appendix*) [75]. The modifications refer to the inclusion of the three-body interaction coefficient [76], which is essential to reproduce the measured concentrations within dense phases [17]. From the fits, the estimated value of the critical volume fraction was found to be $\phi_\mathrm{c} \approx 0.1$. The contour length of each A1-LCD molecule is 137 l.u. To achieve the estimated volume fraction using 200 molecules when the system is quenched to be at the critical volume fraction, the dimensions of the cubic box would have to be $L_\mathrm{sim} \approx 65$ l.u. to a side. However, this box size is less than half the contour length of each chain. This is a problem because in addition to creating spurious interactions between images, simulations in small boxes can only accommodate density fluctuations that are smaller than the contour length of a single chain. Additionally, as ${T \to T_\mathrm{c}}$, the correlation length $\xi_\mathrm{c}$ diverges, and this cannot be captured in small boxes.

To estimate the appropriate numbers of molecules and box sizes, we used the following considerations. For fixed $\phi \approx \phi_\mathrm{c}$, the adequate box length will scale as $L_\mathrm{sim} \propto n^{1/3}$, where $n$ is the number of A1-LCD molecules. To determine the minimum number of molecules that would be required to map phase behaviors close to criticality, the box length $L_\text{sim, target}$ at $\phi \approx \phi_\mathrm{c}$ should be between $1.5\times N$ and $2\times N$, where $N = 137$ is the contour length for A1-LCD. This gives

\begin{equation*} { n_{\mathrm{min}} \; = \; 200 \left(\frac{L_{\mathrm{sim,target}}}{L_{\mathrm{sim},200}}\right)^3, }\end{equation*}

where $L_{\mathrm{sim},200} = 65$ l.u. and $L_{\mathrm{sim,target}} = 1.5\times137\; \mathrm{l.u.}$ or $L_{\mathrm{sim,target}} = 2\times137\; \mathrm{l.u.}$ Substituting these values, we obtain

\begin{equation*} \begin{aligned} {n_{\text{min, 1.5N}}} &amp; = 200\left(\frac{205.5}{65}\right)^3 \!\approx 6.3\times10^3,\\[4pt] {n_{\text{min, 2N}}} &amp; = 200\left(\frac{274}{65}\right)^3 \!\approx 1.5\times10^4. \end{aligned}\end{equation*}

Simulations with ${n = 10^4}$ lie within the prescribed range, and this should be adequate to map the phase behavior in the vicinity of the critical point. In the simulations that access the critical regime, we used a cubic box of size $L_\mathrm{sim} = 240 \;\mathrm{l.u.}$ for a system of $10^4$ A1-LCD molecules.

### 3.2. Assessing finite-size artifacts and established methods for mapping critical points

It is common practice to assume that the order parameter defined by $\Delta \phi = (\phi_\mathrm{dense} - \phi_\mathrm{dilute})$ scales as $\left(\frac{T_\mathrm{c}-T}{T_\mathrm{c}}\right)^\beta$, with $\beta$ set to be ${\approx}0.33$, being the exponent that is expected for the 3D Ising model. Here, $\phi_\mathrm{dense}$ is the volume fraction of the dense phase, $\phi_\mathrm{dilute}$ is the volume fraction of the dilute phase, $T$ is the simulation temperature, and $T_\mathrm{c}$ is the critical temperature. The procedure that is typically followed is to use the law of rectilinear diameters, fix $\beta$ to be 0.33, and use regression analysis to estimate $T_\mathrm{c}$, $\phi_\mathrm{c}$, and a pre-factor $A_0$ [20, 57, 59–61, 77, 78]. Note that the scaling of the order parameter is only valid in the vicinity of the critical point [79]. Accordingly, caution is needed when using approaches based on regression analysis [80].

**Figure 3.**
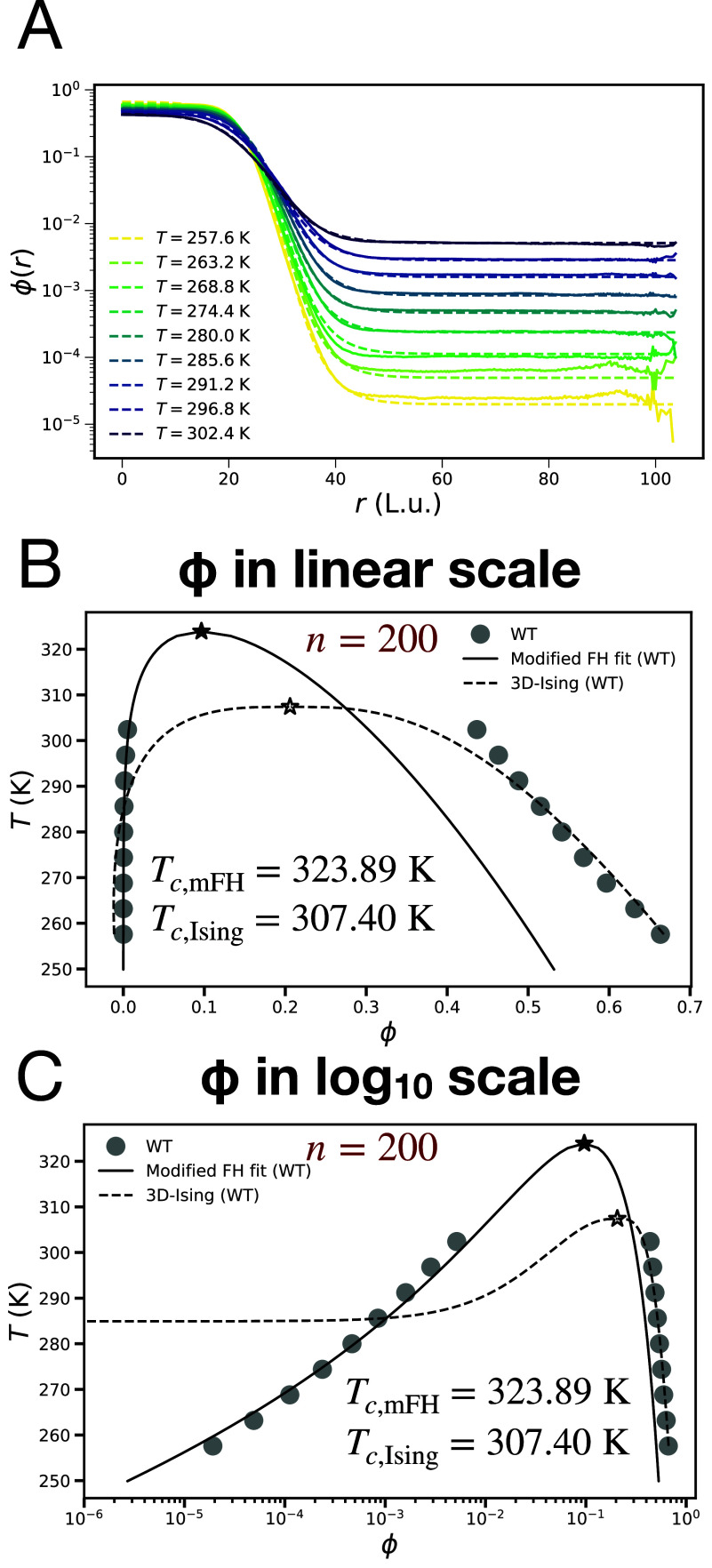
Finite-sized systems pose limitations in mapping the full binodal. The results are shown here for A1-LCD. (A) $T$-dependent radial density profiles $\phi(r)$ for the system containing $200$ molecules of WT A1-LCD. Different profiles were obtained for different temperatures $T$ (in Kelvin). For each temperature, the computed radial density profile was used to extract the dense and dilute coexistence concentrations by fitting them to the Fisk–Widom function. (B)–(C) Comparative analysis of estimates of critical points and fitting binodals to the modified FH model and the 3D-Ising model. The results are shown in (B) linear scale and (C) log scale of the volume fraction $\mathrm{\phi}$.

**Figure 4.**
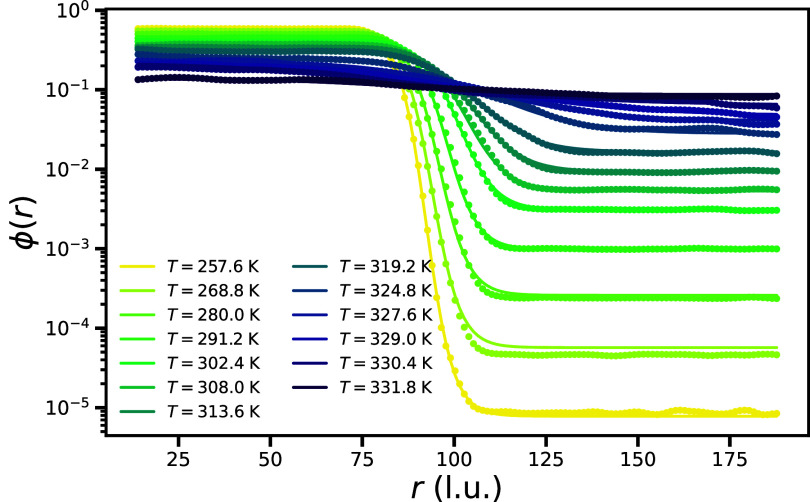
$T$-dependent radial density profiles for a system with $10^4$ molecules of A1-LCD. Radial density profiles $\phi(r)$ as a function of $r$ (in lattice units) at different temperatures for the $10^4$ chain system, which are then fit using the Fisk–Widom function to estimate the coexisting dense and dilute phase concentrations ($\phi_{\mathrm{dense}}$ and $\phi_{\mathrm{dilute}}$).

To assess whether the vicinity of the critical regime can be sampled in simulations of finite-sized systems, we examined the $T$-dependent radial density profiles for a system of 200 A1-LCD molecules (figure 3(A)) and compared these profiles to those obtained using $10^4$ molecules (figure 4). A defining feature of the critical regime is that the concentration of the dilute phase should approach that of the dense phase. Concentrations extracted from these profiles using the Fisk–Widom function [74] (see *SI appendix*) show that at all temperatures sampled, the dilute and dense phase concentrations differ by over an order of magnitude (circles in figures 3(B) and (C)). This suggests a lack of data points around the critical point. The situation cannot be remedied by increasing the simulation temperature in small simulation boxes. In fact, at temperatures above 302.4 K, we observe obtain radial density profiles with large fluctuations across the entire box. This non-physical behavior arises because sampling the critical regime for a simulation with 200 molecules of A1-LCD requires a rather small box of size $L_\mathrm{sim} \approx 65$ l.u. Small simulation boxes create the dual challenge of small numbers of molecules being used to mimic macroscopic systems, and suppression of density fluctuations because the box size is considerably smaller than the correlation length, which will be larger than the contour length of each molecule.

The upshot is that the finite-sized simulations comprising 200 molecules cannot access the critical regime. Hence, the common practice is to enforce the scaling of the order parameter across the entirety of the computed binodal. The reduced temperature is defined as

\begin{equation*} T_\mathrm{r} = 1 - \frac{T}{T_\mathrm{c}},\end{equation*}

where $T_\mathrm{c}$ is the critical temperature. According to the law of rectilinear diameters, the average of the coexisting phase volume fractions varies linearly with temperature:

\begin{equation*} \frac{\phi_{\mathrm{dense}}\left(T\right) + \phi_{\mathrm{dilute}}\left(T\right)}{2} = \phi_\mathrm{c} - m \, T_\mathrm{c} \, T_\mathrm{r},\end{equation*}

where $\phi_\mathrm{c}$ is the critical volume fraction and $m$ is the slope. Next, the difference between the dense and dilute arms is taken to scale as a power law with Ising critical exponent $\beta \approx 0.33$:

\begin{equation*} \Delta \phi\left(T\right) = \phi_{\mathrm{dense}}\left(T\right) - \phi_{\mathrm{dilute}}\left(T\right) = A_o \, T_\mathrm{r}^{\beta},\end{equation*}

where $A_o$ is a non-universal amplitude. Combining these two expressions, the binodal branches are written as

\begin{align*} \phi_{\mathrm{dense}}\left(T\right) &amp; = \phi_\mathrm{c} - m \, T_\mathrm{c} \, T_\mathrm{r} + \tfrac{1}{2} A_o \, T_\mathrm{r}^{\beta},\end{align*}

\begin{align*} \phi_{\mathrm{dilute}}\left(T\right) &amp; = \phi_\mathrm{c} - m \, T_\mathrm{c} \, T_\mathrm{r} - \tfrac{1}{2} A_o \, T_\mathrm{r}^{\beta}.\end{align*}

The system of equations combined with setting $\beta \approx 0.33$, and knowledge of the computed values of $\phi_\mathrm{dense}(T)$ and $\phi_\mathrm{dilute}(T)$ across the entire binodal are used to estimate $m$, $T_\mathrm{c}$, $\phi_\mathrm{c}$, and $A_0$.

We assessed the quality of the fits obtained using the approach laid out above by utilizing the 200 chain A1-LCD system (figures 3(B) and (C)). Details of the fitting procedure may be found in the *SI appendix*. The dilute arm is fit very poorly by imposing the scaling of the order parameter from the 3D Ising model to apply across the entire binodal. This is expected, since the scaling of the order parameter should only apply in the vicinity of the critical point. Next, we fit the computed binodal to a modified Flory–Huggins model [17, 51, 75]. While this method generates a mean field scaling of the order parameter in the vicinity of the critical point [76], its use for estimating the location of the critical regime is of practical utility. We assessed fits obtained using the two methods by computing the exponential root mean square log (ERMSL) [52] to compare the difference between the estimate of the coexisting dilute and dense phase volume fractions generated by fitting to the 3D-Ising model versus the modified Flory–Huggins theory. A value of 10 implies that the estimated volume fractions from the fits are different by an order of magnitude, whereas a value of one implies perfect agreement. The ERMSL values obtained using the 3D-Ising model were 6.96 and 1.1 for the dilute and dense arms, respectively. For fits of the binodal to the modified Flory–Huggins theory, we obtain ERMSL values of 1.27 and 1.34 for the dilute and dense arms, respectively. Overall, we conclude that the critical regime is inadequately sampled or not sampled at all in simulations based on 200 A1-LCD molecules, thus emphasizing the challenges posed by finite size artifacts [81] (figure 3).

### 3.3. Mapping the binodal for large systems and assessing its accuracy

**Figure 5.**
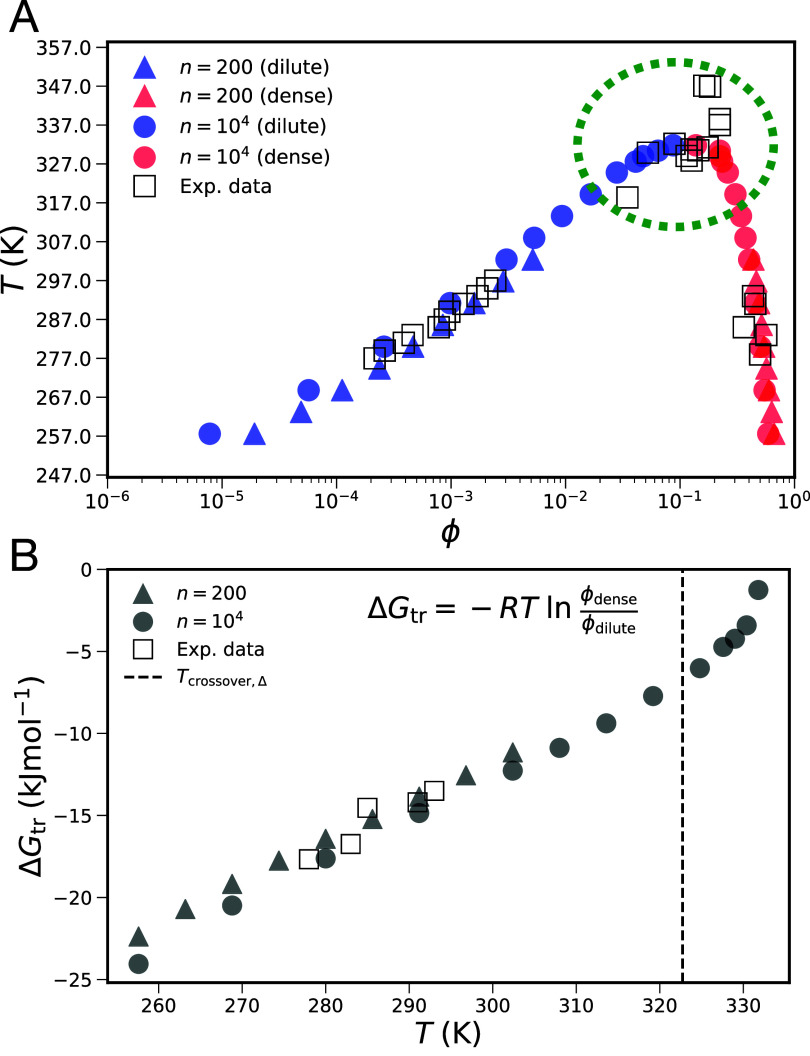
Comparisons between computed and measured binodals. (A) Binodal of A1-LCD, showing coexistence concentrations of the dilute phase (blue) and dense phase (red). Results from simulations with $10^4$ chains are shown as circles, and those from simulations with $200$ chains are shown as triangles. The measured coexistence points [51] are shown as open squares. The critical regime is delineated by the green dotted oval. In this regime, the experiments reveal large density fluctuations. (B) To assess the accuracy of the binodal, we computed transfer free energies, calculated as $\Delta G_{\mathrm{tr}} = -RT \ln \!\left(\phi_{\mathrm{dense}}/\phi_{\mathrm{dilute}}\right)$. These are plotted as a function of temperature. Here, $R = 8.314 \,\mathrm{Jmol}^{-1} \mathrm{K}^{-1}$. Triangles correspond to data from the $200$-chain simulations, circles to data from $10^4$-chain simulations, and squares to measurements. The dashed vertical line corresponds to $T_{\mathrm{crossover},\Delta} \approx 322.74\;\mathrm{K}$ between the mean field and critical regimes.

For the simulations that include $10^4$ A1-LCD molecules, the presence of a two-phase system in the simulation cell is evident from the temperature-specific radial density profiles $\phi(r)$ (figure 4). The simulations for a system with $10^4$ chains show that the envelope of the two-phase regime extends to higher temperatures. As $T$ increases towards the apparent critical regime, we observe nearly equivalent dilute and dense phase volume fractions (figure 5(A)). To assess the accuracy of the computed binodals, we compared them to one another and to experiments. Note that the experiments used absorbance for measuring off-critical coexistence points, and cloud point measurements to map the critical region [51]. While the cloud point measurements are noisy due to large density fluctuations, they suggest that $T_\mathrm{c} > 330$ K. To put the comparisons between computed and measured binodals on a quantitative footing, we computed transfer free energies using $\Delta G_\mathrm{{tr}} = -RT \ln \left(\frac{\phi_{\mathrm{dense}}}{\phi_{\mathrm{dilute}}}\right)$ in units of $\mathrm{kJmol}^{-1}$ [82]. In the regime where the comparisons are feasible, the computed transfer free energies from both the 200-chain and $10^4$-chain systems showed good agreement with the measurements (figure 5(B)). These comparisons give us confidence regarding the accuracy of the model used in the LaSSI simulations.

**Figure 6.**
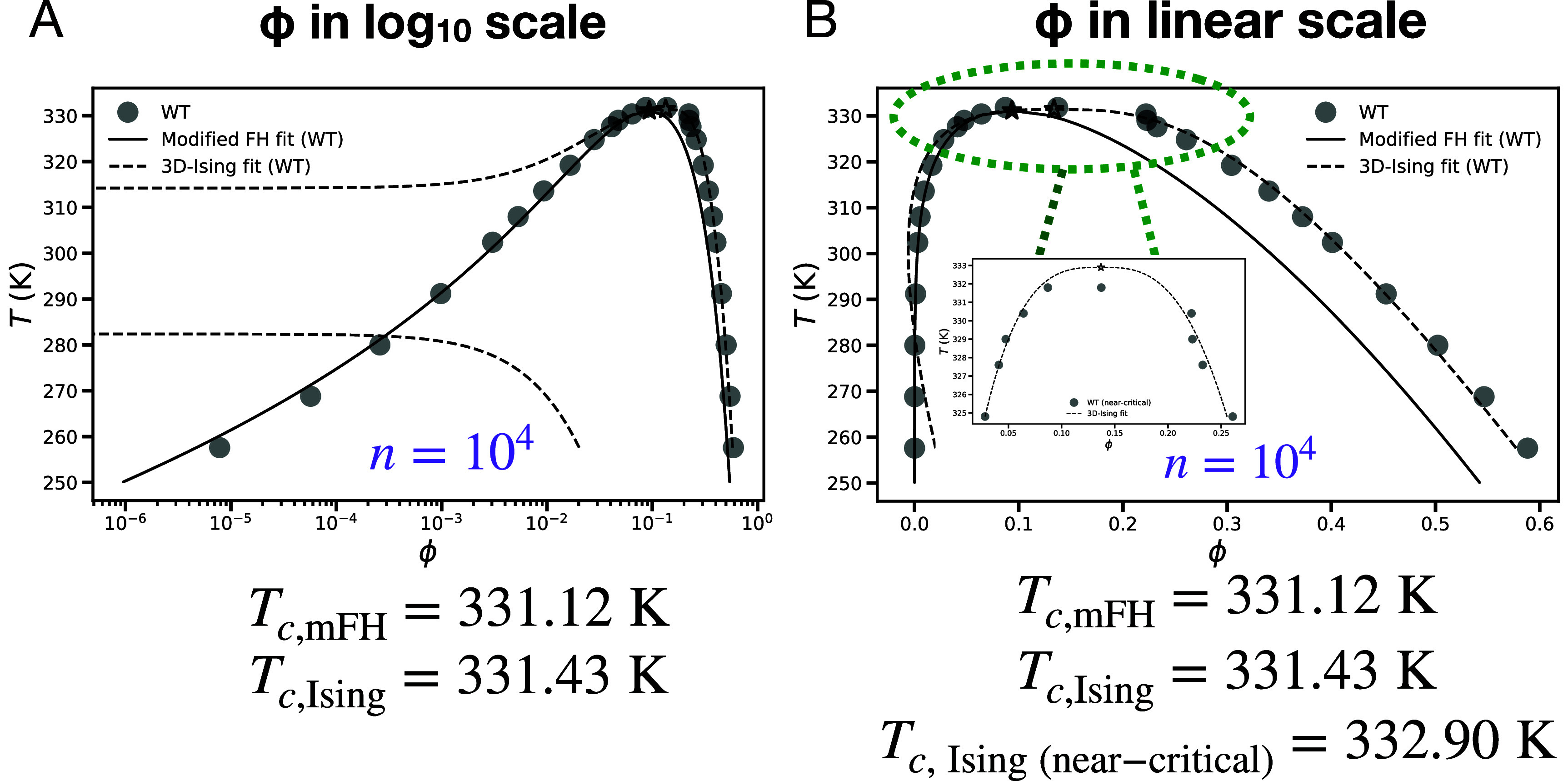
Illustration of the error of using a single model to fit the entire binodal. Comparative analysis of estimates of critical points based on two different approaches. Fits performed using the modified FH model and the 3D-Ising model to the entirety of the binodal for a system comprising $10^4$ molecules of WT A1-LCD. The results are shown in both (A) log scale and (B) linear scale of the volume fraction $\mathrm{\phi}$. The inset in (B) shows results obtained by fitting the 3D-Ising model to points in the critical regime, i.e. $T > T_{\mathrm{crossover},\Delta}$ (see figure 7(B)). Even though the analysis has access to concentrations in the critical regime, the fit to the dilute arm using the 3D-Ising model approach is poor.

**Figure 7.**
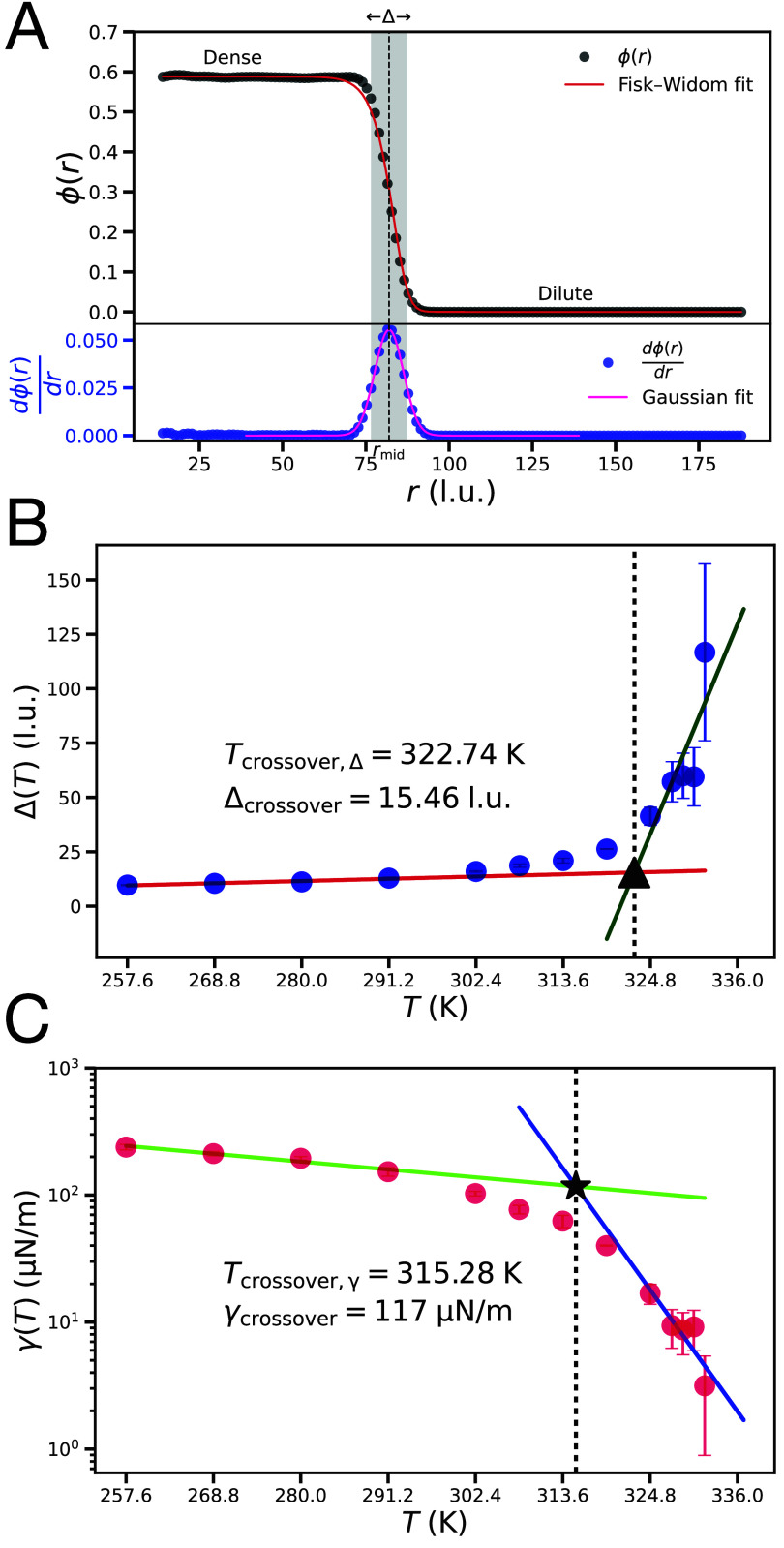
Demarcation of the off-critical (mean field) and near-critical regimes. (A) For a given temperature, the derivative of the radial density profile $\phi(r)$ is fitted to a Gaussian function, from which the interfacial width $\Delta$ is extracted as the full-width-at-half-maximum (FWHM). The midpoint of the interface $r_\mathrm{mid}$ is the mode of the Gaussian. We insert the computed values of $\Delta$ and $r_\mathrm{mid}$ into a hyperbolic tangent Fisk–Widom function [52, 74]. This function is then fit to the $\phi(r)$ profile to estimate the coexisting dense and dilute-phase concentrations ($\phi_{\mathrm{dense}}$ and $\phi_{\mathrm{dilute}}$). (B) Temperature dependence of $\Delta(T)$ showing a crossover from the off-critical to the near-critical regime at $T_{\mathrm{crossover},\Delta} \approx 322.74\;\mathrm{K}$, with a corresponding interface width $\Delta_{\mathrm{crossover}} \approx 15.5$ lattice units. (C) A similar crossover is observed for the interfacial energy per unit area $\gamma(T)$, with $T_{\mathrm{crossover},\gamma} \approx 315.28\;\mathrm{K}$ and $\gamma_{\mathrm{crossover}} \approx 117\,\mathrm{\mu N m^{-1}}$.

Fitting the entire binodal for the $10^4$-chain system to the scaling of the order parameter that is based on the 3D Ising model yields a higher estimate of $T_\mathrm{c}$, when compared to the 200-chain system. However, the reproduction of the binodal is very poor (figure 6) and this is expected because the scaling is invalid away from the critical regime. When we restrict the analysis to the near-critical regime, i.e. to temperatures above $T_{\mathrm{crossover},\Delta}$ (see figure 7(B)), the estimated $T_\mathrm{c}$ does not change much. Even here, the symmetry expected of the order parameter is not present in the actual data, and hence the fit to the binodal in the vicinity of the critical point remains poor (figure 6(B)). We also used the modified Flory–Huggins model to fit the computed binodal. Note that the value of $\beta$ for the scaling of the order parameter will always be 0.5, and this goes against expectations of behaviors in the vicinity of the critical point [76, 83]. Comparisons of the fits to the 3D-Ising model versus the modified Flory–Huggins theory were performed by computing the ERMSL as was done for the 200-chain system. For fits to the 3D-Ising model, we obtained ERMSL values of 46.05 and 1.08 for the dilute and dense arms, respectively. Conversely, for the modified Flory–Huggins theory, we obtained ERMSL values of 1.25 and 1.29 for the dilute and dense arms, respectively.

### 3.4. Delineating the critical and off-critical regimes

At a given temperature $T$, coexisting dense and dilute phases are delineated by an interface of width $\Delta$. In the critical regime, the system is defined by large density fluctuations, and $\Delta$ will diverge as $T \rightarrow T_\mathrm{c}$ [46, 84]. The derivative of $\phi(r)$ with respect to $r$ (figure 7(A)) helps delineate the location of the interface (see *SI appendix*). We inserted the computed values of $\Delta(T)$ and $r_\mathrm{{mid}}(T)$ into a hyperbolic tangent, Fisk–Widom function [52, 74]. This function is then fit to the $T$-dependent profiles for $\phi(r)$ to estimate $\phi_\mathrm{dense}(T)$ and $\phi_\mathrm{dilute}(T)$ (figures 4 and 7(A)).

The width $\Delta(T)$ shows the presence of two regimes as a function of temperature, with the crossover temperature being ${T_{\mathrm{crossover},\Delta} \approx 322.74\;\mathrm{K}}$ (figure 7(B)). Above, $T_{\mathrm{crossover},\Delta}$, $\Delta(T)$ increases rapidly with $T$. This is characteristic of the divergence one expects for the width of the interface as the critical temperature $T_\mathrm{c}$ is approached [46, 84]. Thus, we used ${T_{\mathrm{crossover},\Delta}}$ to demarcate the critical and off-critical (mean field) regimes, with $T > T_{\mathrm{crossover},\Delta}$ designated as the critical regime. The interfacial tension is the free energy penalty associated with increasing the area of the interface and it is computed as $\gamma(T) = \frac{k_\mathrm{B} T} {(l\Delta(T))^{2}}$ (figure 7(C)) [85]. Here, $l = 4$ is a factor that converts 1 $\mathrm{l.u.}$ to 4 $\mathrm{\unicode{x00C5}}$ (see section 2.2 in Methods). The interfacial tension shows a sharp decrease above ${T_{\mathrm{crossover},\Delta}}$ and this is consistent with the expectation that $\gamma(T)$ will approach zero as $T \rightarrow T_\mathrm{c}$ [46]. The calculated values of $\gamma(T)$ are consistent with measured interfacial tension values for condensates [4, 42, 86–88] and for colloid-polymer systems [85].

### Analysis of Binder cumulants to estimate $T_\mathrm{c}$

3.5.

**Figure 8.**
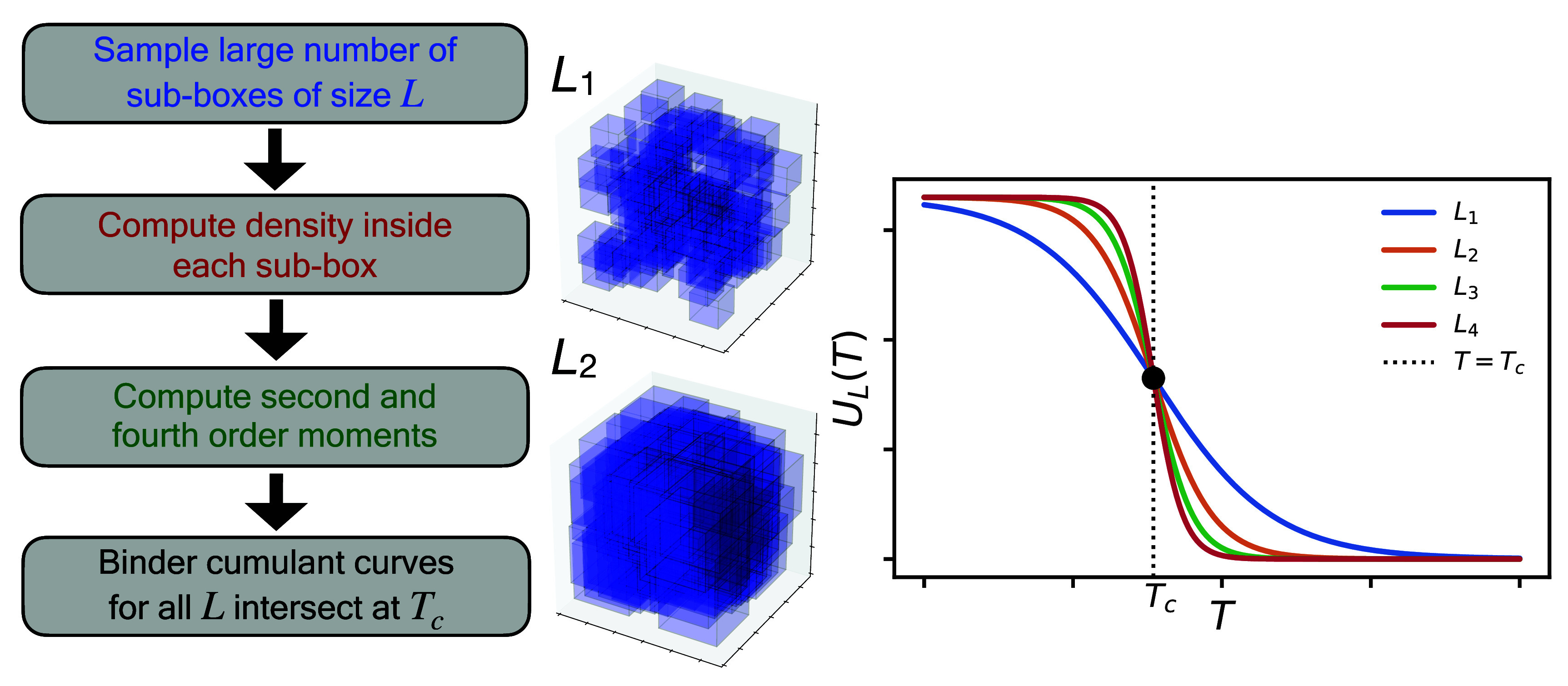
Workflow for Binder cumulant analysis. Schematic illustrating the procedure used to obtain an accurate and independent estimate of the critical temperature $T_\mathrm{c}$. This is based on computations of higher order moments of the order parameter $m_L = \phi_L(T) - \langle \phi_L(T) \rangle$, which quantifies the deviation of the density from its mean value within a cubic sub-box of length $L$ at temperature $T$. A large number of cubic sub-boxes of length $L$ are sampled. The variance and the fourth-order cumulant are obtained from $m_L$ for each of the sampled sub-boxes. The curves for Binder cumulants, computed for different $L$ values, intersect at $T_\mathrm{c}$.

To take advantage of the accuracy afforded by large-scale simulations, we used an approach based on Binder cumulants and rigorous finite-size scaling [64, 71, 81, 89–91]. The Binder cumulants quantify the mean density (first moment), density fluctuations (based on the second moment), and the anisotropic nature of the fluctuations (based on the fourth moment). The finite-size scaling uses cubic sub-boxes, each of a different length $L$. Each sub-box of length $L$ is used to probe the mean density and cumulants on the length scale of the sub-boxes for all temperatures. A schematic of the workflow deployed to calculate and analyze Binder cumulants is shown in figure 8. Note that densities are computed as the number of polymer beads per unit volume.

**Figure 9.**
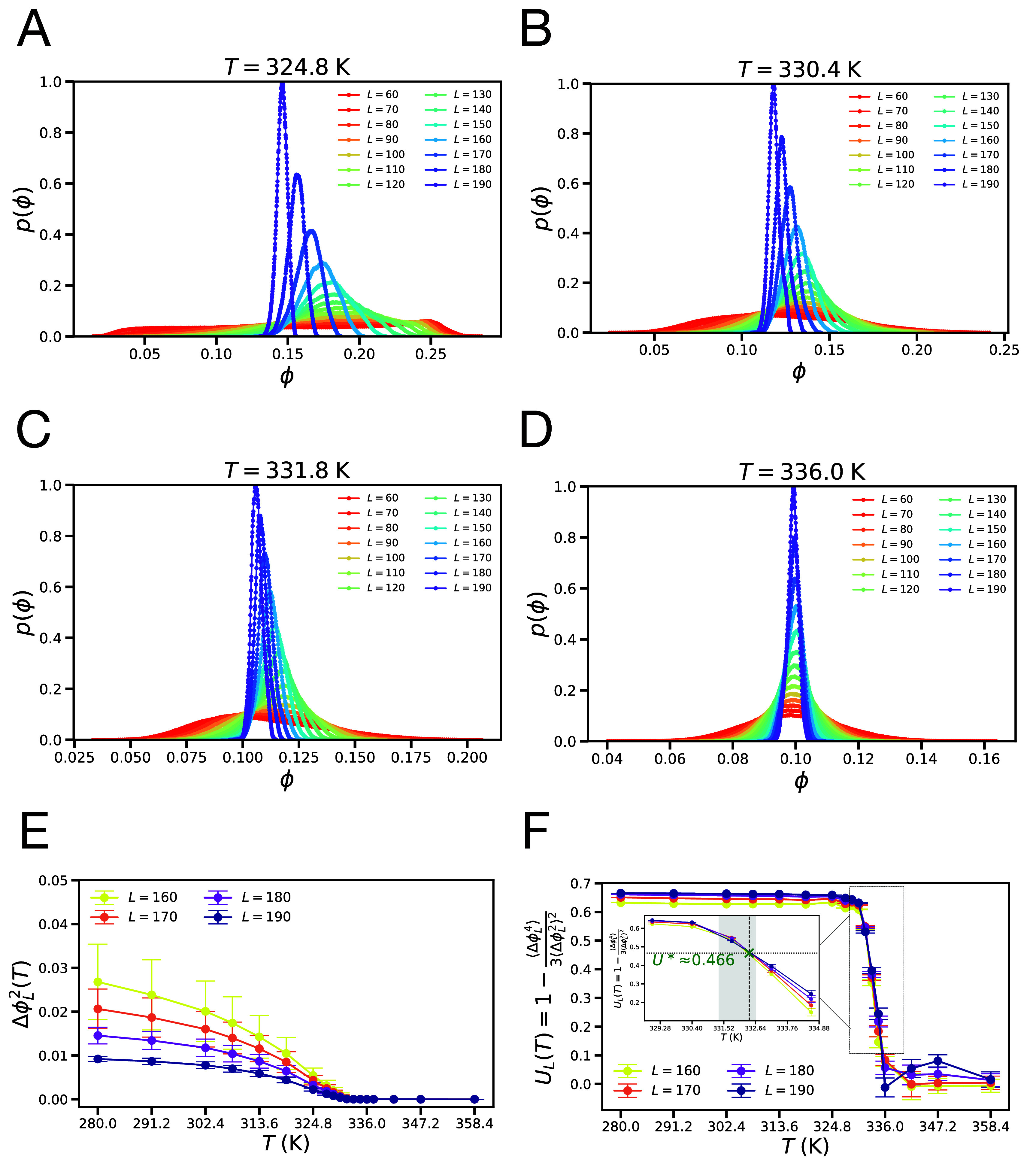
Distributions of the order parameter and results from Binder cumulant analyses.(A)–(D) Probability distributions of the sub-box densities $p(\phi)$ at various temperatures $T$ and for different sub-box sizes $L$ (in lattice units). (E) The variance of the order parameter plotted as a function of the temperature $T$ for various sub-box sizes $L$. (F) Binder cumulant $U_L(T)$ as a function of $T$ for various sub-box sizes $L$. The curves intersect at a median critical temperature of $T_\mathrm{c} \approx 332.42\;\mathrm{K}$, as shown in the inset. The shaded gray region (in the inset) indicates the range between the lower and upper bounds of the pairwise crossing temperatures, and the green cross marks the intersection point corresponding to the median $T_\mathrm{c}$. At the median value of $T_\mathrm{c}$, the value we obtain for $U_L$ corresponds to $U^\ast = 0.466$, which is the value that has been determined for $U_L$ at the $T_\mathrm{c}$ of the 3D Ising model [92].

Probability distributions, $p(\phi)$, calculated within sub-boxes of different size $L$, are shown in figures 9(A)–(D) for four representative temperatures (also see figure S3 in the *SI appendix*). Below $T_\mathrm{c}$, and for small sub-boxes, the density distributions are bimodal figures 9(A)–(C), reflecting the coexistence of dense and dilute phases. The density distributions become unimodal and Gaussian for all sub-box sizes $L$ as $T_\mathrm{c}$ is approached and crossed (figure 9(D)).

For each sub-box of length $L$, the variance was computed using $(\Delta\phi_L(T))^2 = m^2_L(T)$ and the fourth-order cumulant was computed using: $U_L(T) = 1 - \langle m^4_L \rangle / (3 {\langle m^2_L \rangle}^2)$, where $m_L = \phi_L(T) - \langle \phi_L(T) \rangle$ is the order parameter. When the variance and fourth-order cumulant are respectively plotted as a function of $T$ for various sub-box sizes $L$, curves of $m_L^2(T)$ and $U_L(T)$ intersect at a common point that corresponds to the true $T_\mathrm{c}$ (figures 9(E) and (F)). Note that this analysis was performed for cumulants computed in sub-boxes of size $L \unicode{x2A7E} 160\;\mathrm{l.u.}$ This choice ensures that all density fluctuations are fully accounted for in estimating $T_\mathrm{c}$. The mean and median values of $T_\mathrm{c}$ were found to be $332.14\;\mathrm{K}$ and $332.42\;\mathrm{K}$, respectively (figure 9(E) and (F)). At the median value, we find that $U_L(T) \approx 0.466$. This is congruent with the value of $U^\ast = 0.466$ that is expected at $T = T_\mathrm{c}$ for the 3D Ising model [92]. The statistical error in the estimation of $T_\mathrm{c}$ is obtained from the lower and upper bounds of the set of all pairwise crossings of the Binder cumulant curves (shown in the inset of figure 9(F)). The values of these lower and upper bounds are $331.35 \;\mathrm{K}$ and $332.63\;\mathrm{K}$ respectively.

**Figure 10.**
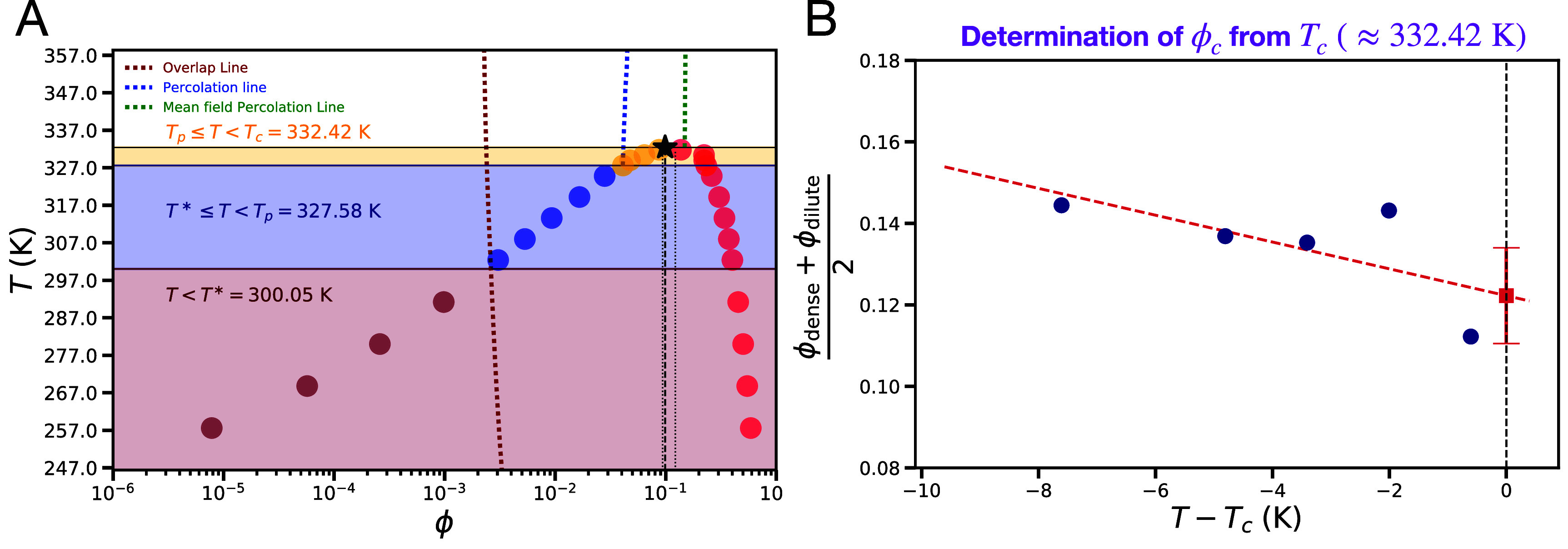
Fully annotated binodal for WT A1-LCD and determination of $\mathrm{\phi_\mathrm{c}}$. (A) Fully annotated binodal for WT A1-LCD. The overlap and the percolation lines are indicated by maroon and blue dashed lines, respectively. The mean field percolation line, mapped using the generalized bond percolation model [21] (see *SI appendix*), is shown as a green dashed line. The overlap and the percolation lines intersect the left arm of the binodal and divide it into three distinct regimes. The percolation threshold shown by the dashed blue line has a value of $\phi_\mathrm{p} = 0.0429$, and it is determined by using sticker-sticker contacts to define clusters. $T_\mathrm{p} = 327.58\;\mathrm{K}$. The critical point ($\phi_\mathrm{c} \approx 0.099, T_\mathrm{c} \approx 332.42\;\mathrm{K}$) is marked by an asterisk. Here, $\phi_\mathrm{c}$ is estimated by a vertical linear extrapolation and identified as the value where the vertical drop from $T_\mathrm{c}$ intersects the abscissa (shown by the dashed black line). We also show the upper and lower bounds in the estimation of $\phi_\mathrm{c}$ using two dotted black lines that correspond to vertical extrapolations done from the upper and lower bounds in the estimation of $T_\mathrm{c}$ (shown in figure 9(F) as the bounds of the shaded gray region). Red circles denote the dense phase points, while the dilute phase is shown by circles whose color corresponds to the regime to which the points belong. These regimes are: (i) the low-temperature regime (maroon), for $T < T^\ast$; (ii) the intermediate regime (navy blue), for $T^\ast \unicode{x2A7D} T < T_\mathrm{p}$; (iii) the high-temperature regime closest to criticality (orange), for $T_\mathrm{p} \unicode{x2A7D} T < T_\mathrm{c}$. (B) An independent estimate of the critical volume fraction $\phi_\mathrm{c}$ was obtained using the law of rectilinear diameters, with $T_\mathrm{c} = 332.42\;\mathrm{K}$ fixed from Binder cumulant analysis. Bootstrap analysis with 1000 resampled datasets yields mean and median estimates of $\phi_\mathrm{c}$ equal to 0.124 and 0.122, respectively, with a standard deviation of 0.012.

From the point at which the horizontal line that intersects the binodal and the ordinate at $T_\mathrm{c}$ we drop a vertical line and estimate $\phi_\mathrm{c}$ as the value where this line intersects the abscissa. This yields a value of $\phi_\mathrm{c} \approx 0.099$ (figure 10(A)). Using the bounds in our estimate of $T_\mathrm{c}$, we obtain values of $\approx 0.094$ and $\approx 0.122$ as the lower and upper bounds on the estimate of $\phi_\mathrm{c}$, respectively. As an independent approach to estimate $\phi_\mathrm{c}$, we used the law of rectilinear diameters (figure 10(B)). This is valid because the interactions are short-range and the system is in 3-dimensions [93, 94]. The temperature-dependent average of the dense and dilute phase volume fraction $\phi_{\mathrm{diam}}(T)$, computed at different temperatures in the near-critical regime, is fit using linear regression and the knowledge of $T_\mathrm{c}$. A limitation of the law of rectilinear diameters is its sensitivity to the number of data points used in the regression near $T_\mathrm{c}$. To quantify the uncertainty in the estimate obtained for $\phi_\mathrm{c}$, we used bootstrap sampling with replacement of the points in the near-critical regime. For each of the $10^3$ resampled datasets, we fixed $T_\mathrm{c} = 332.42\;\mathrm{K}$ and fit $\phi_{\mathrm{diam}}(T) = \tfrac{1}{2}\left[\phi_{\mathrm{dense}}(T) + \phi_{\mathrm{dilute}}(T)\right] = \phi_\mathrm{c} + m\,(T - T_\mathrm{c})$, obtaining $\phi_\mathrm{c}$ as the intercept. This yields mean and median estimates of $\phi_\mathrm{c}$ $\approx 0.124$ and $\approx 0.122$, respectively (figure 10(B)). The reported slope, $m \approx -0.003\;\mathrm{K}^{-1}$, corresponds to the average value obtained across all the bootstrap samples.

Finally, having demarcated the critical and off-critical regimes and determined $T_\mathrm{c}$ and $\phi_\mathrm{c}$ separately, we analyzed the scaling of the order parameter $\Delta \phi^{\prime} = \left(\frac{{\phi_\mathrm{dense}-\phi_\mathrm{dilute}}}{\phi_{c}}\right)$ as a function of the distance from the critical point $(\phi_\mathrm{c}, T_\mathrm{c})$. This is written as: $\Delta \phi^{\prime} = A_o(1-T/T_\mathrm{c})^{\beta}$. A regression analysis aimed at simultaneous extraction of $\beta$ and $A_0$ [63] becomes problematic because of the sparseness of the number of points in the critical regime. Additionally, the order parameter is not symmetric around the critical point. Instead, even in the vicinity of the critical point, the change in the volume fraction along the dilute arm is larger than the change along the dense arm, thus making $\Delta \phi^{\prime}$ asymmetrical about $T_\mathrm{c}$. Given these considerations, we extracted the value of $\beta$ by analyzing the ratio $\frac{\ln(\Delta\phi_i/\Delta\phi_j)}{\ln(\Delta T_i/\Delta T_j)}$ for different pairs of points $(\phi_i, T_i)$ and $(\phi_j, T_j)$. Here, $\Delta\phi_i = (\phi_{\mathrm{dense},i} - \phi_{\mathrm{dilute},i})$ and $\Delta T_i = (T_\mathrm{c}-T_i)$. This approach was inspired by the method of Meng *et al* that was used to quantify scaling exponents for denatured states of proteins [95]. Note that all points $(\phi_i, T_i)$ and $(\phi_j, T_j)$ were drawn from the critical regime defined by $T > T_{\Delta \mathrm{,crossover}}$. This analysis yielded a set of values for $\beta$, the mean and median of which were $\beta_\mathrm{mean} = 0.3245$ and $\beta_\mathrm{median} = 0.3485$, respectively. For the 3D Ising model, $\beta = 0.3264$ [96]. Thus, we conclude that the critical regime of A1-LCD belongs to the same universality class as the 3D Ising model, which is in accord with prior assumptions [20, 46, 57, 59–61, 63, 77, 78, 80].

### 3.6. Locating the percolation line

The bond percolation threshold, $\phi_\mathrm{p}$, is the volume fraction above which A1-LCD molecules, which can cluster via reversible, physical crosslinks, also form a system-spanning network. We computed the threshold volume fraction $\phi_\mathrm{p}$ for temperatures above and up to $T_\mathrm{c}$. The locus of $\phi_\mathrm{p}$ values is the percolation line (see figure S5 in *SI appendix*, figure 10(A)). When we define clusters using sticker-sticker contacts, we obtain a bond percolation threshold of $\phi_\mathrm{p} = 0.0429$ and a percolation temperature of $T_\mathrm{p} = 327.58\;\mathrm{K}$. If we define clusters based on all beads along each chain, we obtain an estimate of $\phi_\mathrm{p} = 0.0270$ and $T_\mathrm{p} = 324.30\;\mathrm{K}$. For comparison, we performed simulations for athermal, purely repulsive versions of A1-LCD. For clusters defined using sticker-sticker contacts, we obtain a percolation threshold of $\phi_\mathrm{p} = 0.0603$. In contrast, for the athermal, purely repulsive system, if we define clusters based on all beads along each chain, we obtain an estimate of $\phi_\mathrm{p} = 0.0256$ (see figure S6 in *SI appendix*). These comparisons show that the stickers provide specificity in clustering, which can be masked if all beads are included in the definition of clusters. We also used a mean-field bond percolation model [21] to compute the percolation line. This model ignores chain connectivity and correlations (see *SI appendix*). The mean field percolation line intersects the binodal at $T_\mathrm{c}$, and this is consistent with mean field models for PSCP [30]. However, in accord with previous studies [38, 97], we find that the actual percolation line, computed via rigorous clustering analysis [27, 98], intersects the binodal at temperatures that lie below $T_\mathrm{c}$, with the precise value of $T_\mathrm{p}$ being dependent on the method used to define clusters.

### 3.7. The overlap line

Next, we quantified the overlap concentration $\phi^\ast$ as a function of $T$. Above the overlap concentration, the polymer solution is semidilute, and the likelihood of polymers interacting via intermolecular interactions is higher than the likelihood of them interacting via intramolecular interactions [40, 83]. The overlap line is the locus of $\phi^\ast$ values computed as a function of $T$ (see figure S7 in *SI appendix* and figure 10(A). We find that the overlap line intersects the left arm of the binodal at a temperature we designate as $T^\ast$. For A1-LCD, $T^\ast \approx 300.05 \;\mathrm{K}$. Thus, for $T^\ast \unicode{x2A7D} T < T_\mathrm{c}$, the dilute phase is actually a semidilute solution [40, 76, 83].

### 3.8. The binodal has three regimes

Given that the overlap and percolation lines intersect the left arm of the binodal (figure 10(A)), it follows that the binodal is demarcated into three different regimes. These are: Regime I defined by $T < T^\ast$, Regime II defined by $T^\ast \unicode{x2A7D} T < T_\mathrm{p}$, and Regime III defined by $T_\mathrm{p} \unicode{x2A7D} T < T_\mathrm{c}$ (figure 10(A)).

### 3.9. The dense phase is a percolated network

Across the three regimes of the binodal, the concentration of the dense phase changes by a factor of roughly four. Direct calculations of the percolation line and the full binodal show that the right arm of the binodal, which is the locus of $\phi_\mathrm{dense}$ values, is located to the right of the percolation line. This is true irrespective of the approach we use to compute the percolation threshold. Thus, $\phi_\mathrm{dense} > \phi_\mathrm{p}$ for all $T < T_\mathrm{c}$. That $\phi_\mathrm{dense} > \phi_\mathrm{p}$ for all $T < T_\mathrm{c}$ clearly indicates that the phase transition of A1-LCD is consistent with PSCP [51, 52].

### 3.10. Clustering in dilute phases is different across the regimes

In contrast to the dense phase, the concentration of the coexisting dilute phase decreases by more than four orders of magnitude between $T_\mathrm{c}$ and the lowest temperature for which phase behavior was investigated. Demarcation of the binodal into three regimes derives from the intersection of the dilute arm of the binodal by the overlap and percolation lines. Thus, we reasoned that properties of coexisting dilute phases must differ across the three regimes. To test this hypothesis, we analyzed cluster-size distributions in coexisting dilute phases as a function of the temperature $T$.

**Figure 11.**
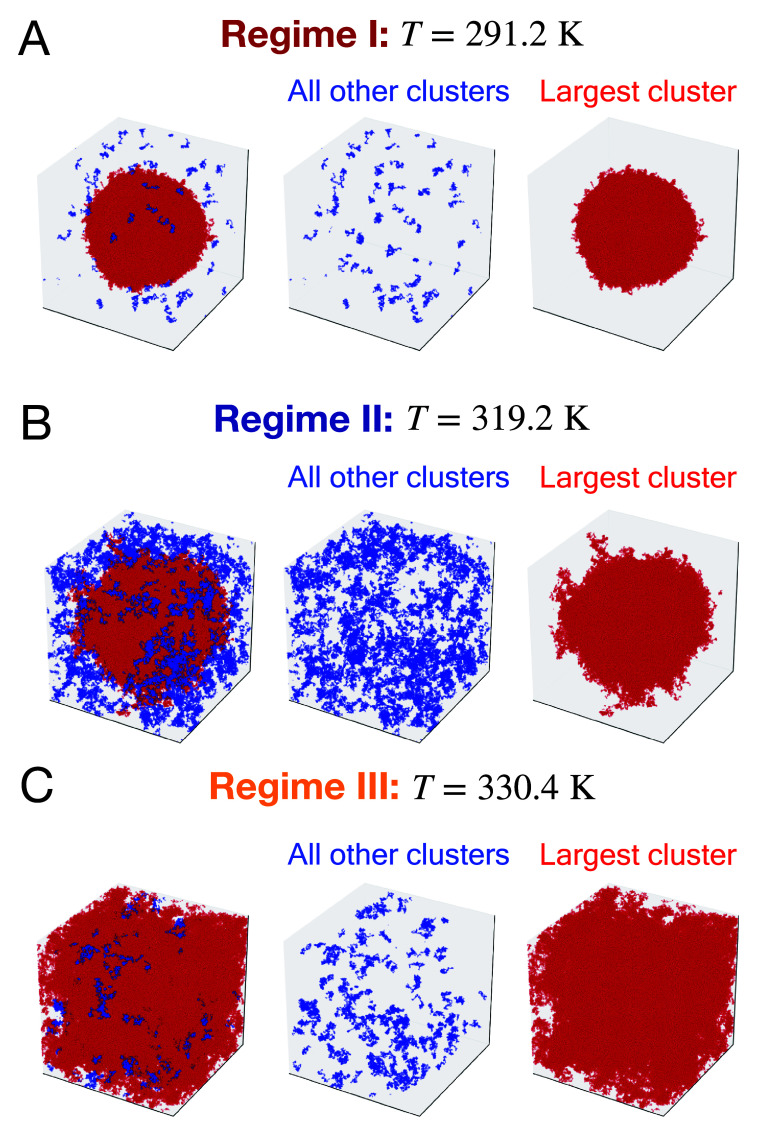
Snapshots showing the coexisting dense and dilute phases in the three distinct regimes of the binodal. (A)–(C) Snapshots showing the largest connected cluster (red) and all other clusters (blue). (A) Regime I of the binodal defined by $T < T^\ast$. The dense phase that is representative of the largest connected cluster is colored in red, while the dilute phase is shown in blue. (B) Regime II of the binodal defined by $T^\ast \unicode{x2A7D} T < T_\mathrm{p}$. Here also, the dense phase that is representative of the largest connected cluster is colored in red, while the dilute phase is shown in blue. (C) Regime III of the binodal defined by $T_\mathrm{p} \unicode{x2A7D} T < T_\mathrm{c}$. Here, the snapshot shows the largest connected cluster (red) and all other clusters (blue) that form. Here, the delineation of dilute and dense phases becomes difficult because the dense phase is no longer confined and swells to become system-spanning.

**Figure 12.**
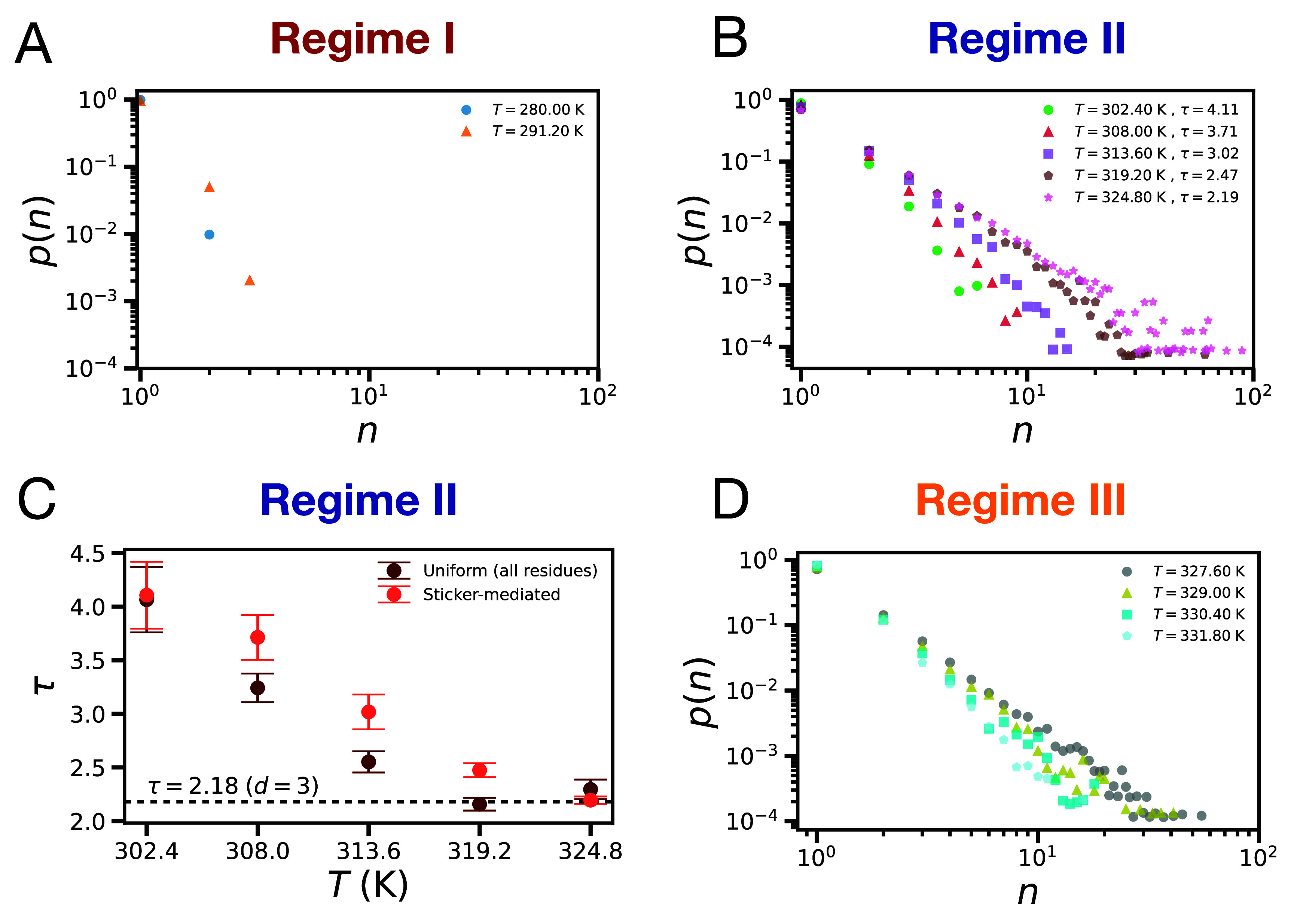
Cluster-size distributions in coexisting dilute phases across the three distinct regimes of the binodal. (A) Cluster size distributions in Regime I, defined by $T < T^\ast$. (B) Cluster size distributions in Regime II, defined by $T^\ast \unicode{x2A7D} T < T_\mathrm{p}$. The legend reports the values of the Fisher exponent $\tau$ obtained from the cluster size distributions of the dilute phase at each temperature in Regime II. (C) Temperature dependence of the Fisher exponent $\tau$ in Regime II for two definitions of connectivity: uniform (all residues) and sticker-mediated. At $T = 324.80\;\mathrm{K}$, $\tau \approx 2.18$ (black dashed line), consistent with the universal value for three-dimensional percolation ($d = 3$). The analysis of $\tau$ is restricted to Regime II, which exhibits broad and heterogeneous cluster size distributions. In Regime I, cluster size distributions in the coexisting dilute phases are dominated by monomers and dimers, yielding an insufficient range for reliable power-law fits. In Regime III, the distinction between dilute and dense phases becomes ambiguous due to system-spanning connectivity. (D) Cluster size distributions in Regime III, defined by $T_\mathrm{p} \unicode{x2A7D} T < T_\mathrm{c}$. For panels (A), (B), and (D), clustering is defined using sticker-mediated connectivity. Cluster size distributions were computed from the final 10 frames of equilibrated simulations and across three independent replicates for each condition.

In Regime I, the cluster-size distribution in the coexisting dilute phase decays steeply as $n$ increases. Essentially, the dilute phase is akin to a vapor of unassociated polymers or clusters comprising at most three molecules (figures 11(A) and 12(A)). In Regime II, the coexisting dilute phase is a semidilute solution [40, 76, 83]. Individual A1-LCD molecules can overlap with one another, forming finite-sized clusters in the dilute phase (figures 11(B) and 12(B)). Here, the probability $p(n)$ of forming clusters containing $n$ molecules can be described using a power-law relation of the form: $p(n) = n^{-\tau}$ [99]. The magnitude of $\tau$ is a measure of the extent of clustering. Higher values of $\tau$ are indicative of smaller clusters being formed, whereas smaller values of $\tau$ imply the converse. We analyzed cluster-size distributions in the coexisting dilute phases as a function of temperature. Using linear regression analysis, which required the fitting of $\log_{10}{p(n)}$ to $-\tau \log_{10}{n}$, we extracted the temperature dependence of $\tau$ (figure 12(C)). The error bars in figure 12(C) correspond to the standard errors from the covariance of the fitted slope. The cluster-size distributions are characterized by exponents that decrease from $\tau = 4.11$ for $T\approx T^\ast$ to $\tau\approx2.18$ for $T \approx T_\mathrm{p}$ (figures 12(B) and (C)).

As the concentration of the coexisting dilute phase increases above the overlap line, cluster formation in the dilute phase becomes easier, as evidenced by the decreasing values of $\tau$ (figures 12(B) and (C)). As a result, larger clusters form, and their abundance, quantified by the values of $p(n)$, increases with increasing $T$. In addition to the formation of heterogeneous distributions of clusters in the dilute phase, Regime II is characterized by an order of magnitude decrease in the interfacial tension which goes from $\approx 100 \,\mathrm{\mu N m^{-1}}$ at $T\approx T^\ast$ to less than $10 \,\mathrm{\mu N m^{-1}}$ as $T_\mathrm{p}$ is approached.

**Figure 13.**
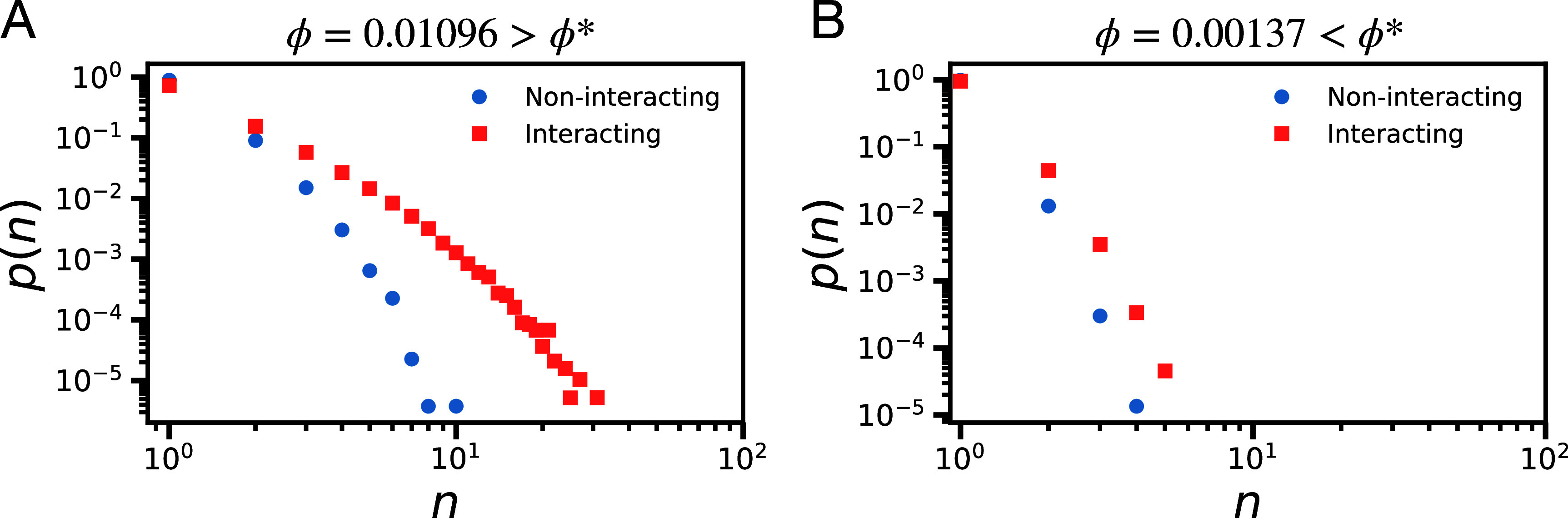
Comparison of the cluster-size distributions obtained for a system of A1-LCD chains versus a non-interacting equivalent of A1-LCD. The interacting system (in this case, A1-LCD) has the pairwise interaction energies as shown in figure 1(D), and the non-interacting equivalent is a version of A1-LCD with all pairwise attractions zeroed out, at the same volume fraction and temperature. The non-interacting system is athermal. For both the systems, simulations were performed at volume fractions that lie in the one-phase regime. Clustering of chains was quantified using a sticker-mediated connectivity. (A) Here, $\phi = 0.01096 > \phi^\ast$ (above the overlap volume fraction). The interacting system (orange) shows a broad cluster size distribution extending to $n \approx 30$, while the non-interacting control (blue) extends upto $n \approx 10$. The excess clustering in the interacting system can be attributed to energetically driven sticker-sticker associations. (B) Here, $\phi = 0.00137 < \phi^\ast$ (below the overlap volume fraction). Both the systems show nearly identical, narrow distributions confined to $n \unicode{x2A7D} 5$, confirming that the contributions from associative interactions at volume fractions below the overlap threshold is almost negligible. All cluster-size distributions were computed from the final 10 frames of the equilibrated simulations at $T = 319.2\;\mathrm{K}$, and across three independent replicates for each condition.

We sought to dissect the generic versus system-specific contributions to the cluster-size distributions observed in Regime II. For this, we fixed the temperature at 319.2 K and quantified the cluster-size distributions in the one-phase regime, choosing concentrations to the left of the dilute arm of the binodal. At a temperature of 319.2 K, the coexisting dilute phase volume fraction is $\phi_\mathrm{sat} = 0.0166$ and the overlap volume fraction is $\phi^\ast = 0.0025$. Thus, $\phi_\mathrm{sat} > \phi^\ast$. We analyzed the cluster-size distributions in the one phase regime for two values of $\phi$ *viz.,* $\phi = 0.00137 < \phi^\ast$ and $\phi = 0.01096 > \phi^\ast$. For each of these volume fractions, we performed two sets of simulations. In the first set of simulations, all attractive interactions between polymer beads were zeroed out. The second set of simulations was based on the full interaction model. The results show that for $\phi > \phi^\ast$, even when attractions are zeroed out and the interactions are purely repulsive, the cluster-size distribution has a power law form, with clusters up to ten molecules forming with finite probability (figure 13(A)). This cluster-size distribution shows a rightward shift when we include the full set of interactions (figure 13(A)). In contrast, for $\phi < \phi^\ast$, the cluster-size distribution shows a sharp decay akin to that of a vapor (figure 13(B)), and this is true for both models. Thus, the key determinant of clustering in Regime II is that the coexisting dilute phase is above the overlap concentration. This provides a generic driving force for clustering, which is further enhanced by attractions between the molecules. It is worth noting that even for flexible homopolymers, the Gaussian cluster theory of Raos and Allegra [100], which incorporates the effects of chain conformations and chain clustering in calculations of phase diagrams [101], shows that the overlap line intersects the left arm of the binodal at temperatures below $T_\mathrm{c}$. Thus, our findings are likely to be generally applicable, as the clustering in dilute phases is governed mainly by whether and where the overlap line intersects the left arm of the binodal.

In Regimes I and II, the dense phases can be described as ‘confined physical gels’ [30]. This is because $\phi_\mathrm{dense} > \phi_\mathrm{p}$, and the single largest cluster localizes to the dense phase (figures 11(A) and (B)) across the range of temperatures corresponding to the two regimes. Note that the interfacial tension $\gamma$ is highest in Regime I and decreases to $\approx 10 \mathrm\,{\mu N m^{-1}}$ at the highest temperature within Regime II. Thus, capillary fluctuations are expected to increase as the interfacial tension decreases [85]. This in turn will weaken the confinement of the percolated network.

Bond percolation is a continuous networking transition that is different from density transitions [9, 28, 39, 98, 102]. It is defined by a percolation threshold, and in 3 dimensions, $\tau = \tau_p = 2.18$ is the Fisher exponent that defines the percolation threshold [98]. As $T$ approaches $T_\mathrm{p}$, we find that $\tau$ approaches and crosses the Fisher exponent, $\tau_p = 2.18$ (figure 12(C)). The mapping of the percolation line was performed without consideration of the critical exponent that defines the percolation threshold. Thus, analysis of the cluster-size distribution provides an independent corroboration of $T_\mathrm{p}$ being the temperature where the percolation threshold is crossed.

In Regime III, both the dense and dilute phases become system-spanning (figure 11(C)). Due to the large density fluctuations and low interfacial tension, the dense phase percolated network is no longer confined. It swells to become system-spanning. This swelling of the dense phase network is concordant with observations made by Smokers and Spruijt from their analysis of how volumes of dense phases change as the critical point is approached and crossed [47]. Above $T_\mathrm{p}$, the percolated networks corresponding to the dense and dilute phases swell and become interconnected (figure 11(C)).

### 3.11. Estimating the theta temperature

The critical point $(\phi_\mathrm{c},T_\mathrm{c})$ marks the end of the two-phase regime, implying that the system enters the one-phase regime for $T > T_\mathrm{c}$. Thus, for $T < T_\mathrm{c}$, the two-body interaction coefficient ($B_2$) is expected to be negative [83]. $B_2$ being negative would imply that the effective interactions between pairs of chains or pairs of residues within a chain are attractive for $T < T_\mathrm{c}$. The theta temperature ($T_\theta$) is the temperature at which $B_2 = 0$, and therefore for a system such as A1-LCD, which has an upper critical solution temperature, it follows that $T_\theta$ should be greater than $T_\mathrm{c}$ [76, 83]. Accordingly, we estimated $T_\theta$ and asked if it follows canonical expectations.

**Figure 14.**
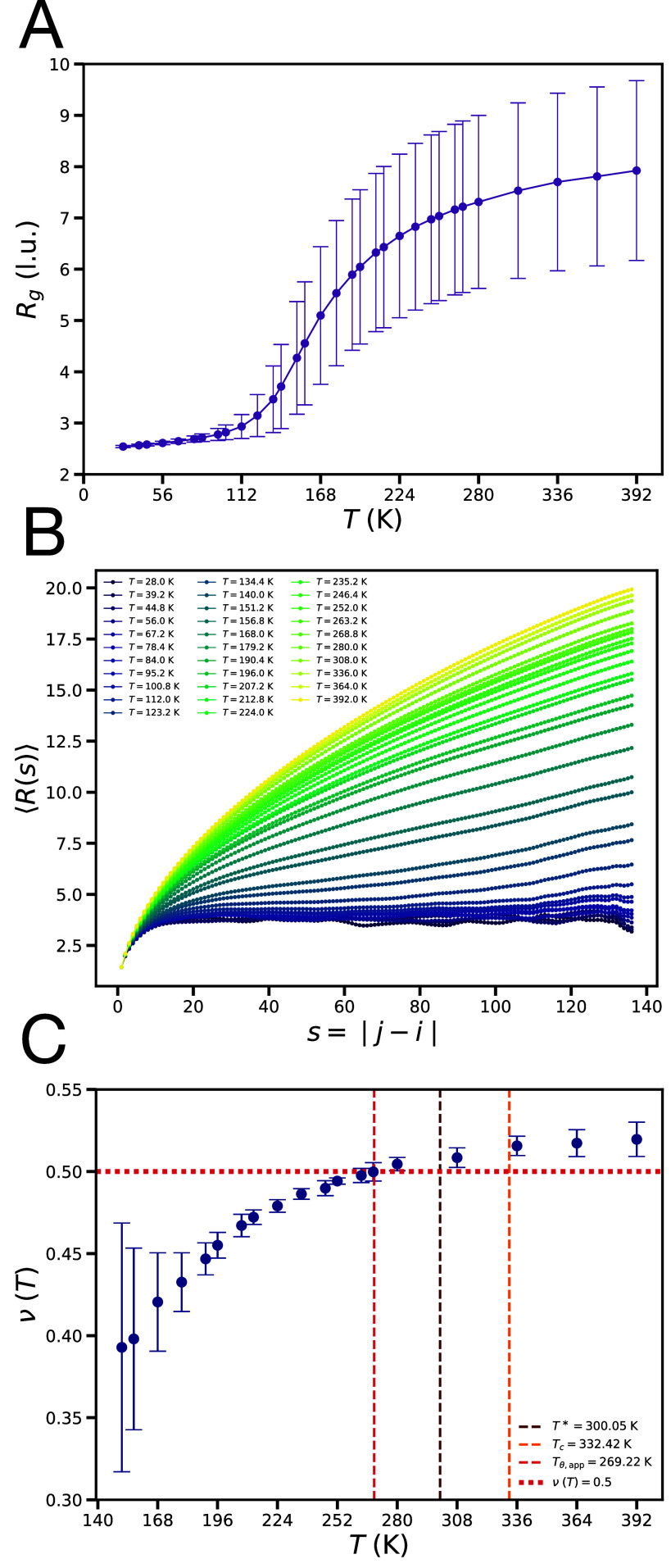
Analysis based on internal scaling profiles severely underestimates the theta temperature ($T_\theta$) for A1-LCD. (A) Radius of gyration ($R_\mathrm{g}$) values from single chain simulations of A1-LCD as a function of the temperature $T$. These were computed from the last 25% of the snapshots from the equilibrated simulations and across three replicates for each temperature. Error bars denote the standard deviation in the computed $R_\mathrm{g}$ for each temperature. (B) $R(s)$ versus $s$ for single A1-LCD chains at various temperatures $T$. (C) $\nu$ values obtained from analysis of the scaling of $R(s)$ versus $s$ as a function of $T$. The $\nu$ values were extracted as the slopes of plots of $\ln(R(s))$ against $\ln(s)$. This analysis was restricted to temperatures where fractal behavior was evident and not for profiles where $R(s)$ is essentially flat because this corresponds to the globule regime. The inferred (apparent) theta temperature $T_{\theta,\mathrm{app}}$, corresponding to where $\nu(T) = 0.5$, is approximately $269.22 \;\mathrm{K}$ (annotated by the red vertical dashed line). This is well below the critical temperature $T_\mathrm{c} \approx 332.42\;\mathrm{K}$. The overlap temperature $T^\ast$ and the critical temperature $T_\mathrm{c}$ are annotated by maroon and orange vertical dashed lines, respectively.

In Flory-style two-parameter theories for homopolymers [83], $T_\theta$ is a simple Gaussian fixed point [103]. Accordingly, $T_\theta$ is the temperature where $B_2 = 0$ for a pair of residues in a chain, and this is taken to be congruent with the temperature at which an individual polymer in a dilute solution behaves like a Gaussian chain. For a Gaussian chain, $R(s) {{\sim}} s^{0.5}$, where $R(s)$ quantifies the ensemble-averaged spatial separation $R(s)$ for all linear segments of length $s$. To identify the lowest temperature at which Gaussian chain statistics are observed, we performed simulations of individual A1-LCD molecules as a function of $T$ (figure 14(A)). We analyzed the scaling of $R(s)$ with $s$ (figure 14(B)). These plots are referred to as internal scaling profiles [104]. If the scaling of $R(s)$ against $s$ shows power law behavior, then $R(s) {{\sim}} s^\nu$.

From the analysis of the internal scaling profiles extracted at different temperatures, we can identify the lowest temperature at which $\nu = 0.5$ (figure 14(C)). We find that the lowest temperature ($T_{\theta,\mathrm{app}}$) at which $\nu = 0.5$ is 269.22 K. Other approaches [105, 106] introduced in the literature yield similar low estimates of $T_{\theta,\mathrm{app}}$. These estimates of $T_\theta$ are incorrect because they are well below the $T_\mathrm{c}$ value of 332.42 K. That the scaling analysis underestimates $T_\theta$ and that the temperature where $B_2 = 0$ will be higher than the $T_{\theta,\mathrm{app}}$ derived from scaling analysis was established by Kholodenko and Freed via conformational space renormalization group calculations [107].

**Figure 15.**
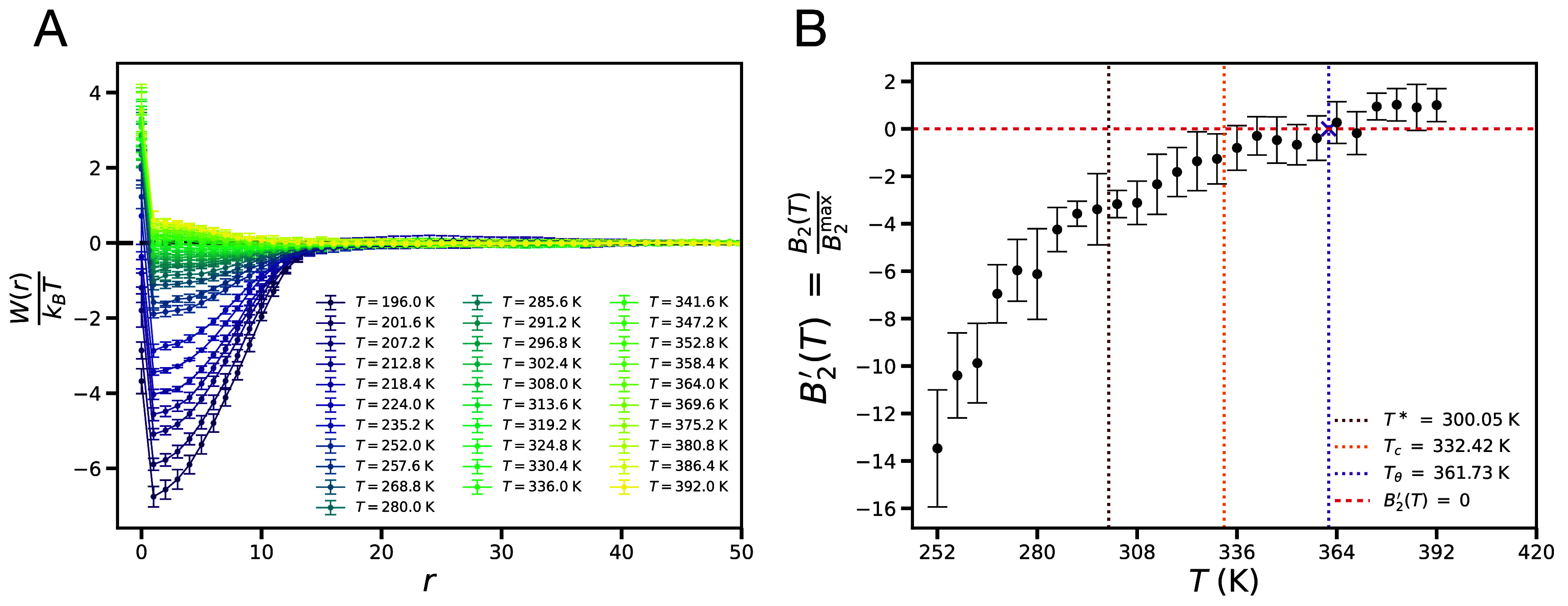
Estimation of the theta temperature ($T_\theta$) for A1-LCD. (A) The potential of mean force $W(r)$ as a function of the separation between the centers-of-mass of two A1-LCD chains, for various temperatures $T$, extracted from umbrella sampling simulations (see Methods section and figure S10 in *SI appendix*) using the Weighted Histogram Analysis Method (WHAM) [108–110]. For each temperature, umbrella sampling simulations were performed across 6 replicates. (B) The normalized two-body interaction coefficient $B_2^{^{\prime}}(T)$ as a function of $T$, obtained from the 2-chain PMF values. Here, $B_2^{^{\prime}}(T) = B_2/B_2^\mathrm{max}$, where $B_2^\mathrm{max}$ is the value of $B_2$ at the highest temperature used in our simulations ($T_\mathrm{max} = 392\;\mathrm{K}$). The temperature at which $B_2^{^{\prime}}(T) = 0$ is the theta temperature ($T_\theta$). From the temperature dependence of $B_2^{^{\prime}}(T)$, we obtain the theta temperature $T_\theta$ using linear interpolation to be $\approx 361.73\;\mathrm{K}$. The overlap temperature $T^\ast$, the critical temperature $T_\mathrm{c}$ and the theta temperature $T_\theta$ are annotated by maroon, orange and purple dotted lines, respectively.

To compute $T_\theta$ directly, we used potentials of mean force (PMFs) $W(r)$ for a pair of A1-LCD molecules at different temperatures $T$ (figure 15(A)). Each PMF quantifies the work done at a given temperature to bring a pair of chains from infinite separation to a distance $r$ between their centers-of-mass. The Mayer $f$-function can be written as $f(r) = \exp(-\beta W(r)) - 1$ and the integral of $-f(r)$ yields the excluded volume, which is used to compute $B_2$ (see *SI appendix*). The normalized $B_2$ crosses zero at a temperature of 361.73 K. This value is greater than $T_\mathrm{c} = $ 332.42 K and appears to be a more reasonable estimate of $T_\theta$ (figure 15(B)).

## 4. Discussion

Mapping of critical points is essential for a complete physical description of the phase behaviors of IDPs such as PLCDs. Knowledge of $T_\mathrm{c}$ provides a direct route to obtaining comparative assessments of sequence-specific driving forces for phase transitions [60, 61]. In this work, we demonstrate that rigorous finite-size scaling methods can be used to map the critical regime. With the full binodal in hand, we asked where the overlap and percolation lines intersect the binodal. We find that these lines intersect the left arm of the binodal thereby setting up three distinct regimes for the coexisting dilute phases [38]. We uncovered distinct features regarding the cluster-size distributions in coexisting dilute phases that are characteristic to each of the three regimes of the binodal (figures 10(A) and 12). The overlap line intersects the low concentration arm of the binodal at a temperature designated as $T^*$ that is well below $T_\mathrm{c}$ for A1-LCD. As a result, the coexisting dilute phase is defined by cluster-size distributions that arise from a combination of the coexisting dilute phase being semidilute and the contributions of intermolecular attractions in semidilute solutions. Bremer *et al* [51] designed and characterized a series of sequence variants of A1-LCD. While the left arms of the binodals vary significantly, the dense phase concentrations change minimally for these variants. These features were reproduced by the LaSSI simulations of Farag *et al* [52] that used 200 molecules for each of the variants. While these simulations do not access the variant-specific critical points, the computed binodals can be fit to the modified Flory–Huggins model [75] (figure S11). The results of the fits show the following trends: Mutations that weaken the driving forces for phase separation are characterized by rightward shifts of the dilute arm of the binodal and lowering of $T_{\mathrm{c,mFH}}$, which is the $T_\mathrm{c}$ estimated using the modified Flory–Huggins model. Data for five variants were analyzed. These variants were chosen because they span the full range in terms of driving forces for phase separation. In all five cases, there exists a putative Regime II, defined by the intersection of the overlap line and the dilute arm of the binodal. This analysis provides a prescription for analyzing results from simulations comprising $O(10^2)$ molecules, which are the norm in the literature. The computed binodal can be fit using the modified Flory–Huggins model to obtain an estimate of $T_{\mathrm{c,mFH}}$ and $\phi_{\mathrm{c,mFH}}$. The location of Regime II can be identified as the region that lies between $T^*$ and $T_{\mathrm{c,mFH}}$. Larger-scale simulations can then be deployed to extract the variant-specific, and temperature-dependent cluster-size distributions in Regime II.

We delineated the mean field and critical regimes by calculating $T_{\mathrm{crossover},\Delta}$. We note that $T_{\mathrm{crossover},\Delta}$ is close in value to $T_\mathrm{p}$. This raises the possibility that widening of the interface might be due to increased proximity to the percolation threshold or vice versa. Certainly, the increased clustering will likely contribute to changing the properties of the interface. The possibility of there being a relationship between $T_{\mathrm{crossover},\Delta,}$ and $T_\mathrm{p}$ merits closer scrutiny through systematic investigations across different systems.

In a recent study, Varma *et al* [111] proposed a connection between the partition coefficient ($K_\mathrm{P}$) and a normalized temperature distance ($t$) from the critical point. We propose that a more direct assessment of the vicinity to the critical point will come from measurements of cluster-size distributions in coexisting dilute phases. This is feasible in cells using super-resolution microscopy [112–116] and *in vitro* using a range of methods [53, 117–124]. Temperatures are useful proxies for the normalized inter-chain interaction coefficients ($B_2$) because $T \propto \frac{1}{\mid B_2\mid}$ [6, 40, 61]. This is relevant for understanding the effects of changes to $B_2$ on clustering in dilute phases. For example, Lan *et al* [113] reported that in unstressed cells, the negative elongation factor (NELF) forms nucleoplasmic clusters that are consistent with what we observe for A1-LCD in the dilute phases of Regime II. Conversely, in stressed cells, the phosphorylation of NELF leads to the formation of macrophases. Setting $B_2^\ast$ to be the value of the interaction coefficient that corresponds to $T^\ast$, we would propose that $\mid\!B_2\!\mid < \mid\!B^\ast_{2}\!\mid$ ($T > T^\ast$) for both unphosphorylated NELF and phosphorylated NELF. At endogenous expression levels, unphosphorylated NELF is in the one-phase regime where it forms clusters corresponding to Regime II. Conversely, phosphorylation moves the system into the two-phase regime by increasing the magnitude of $B_2$ (making $T$ lower), and the coexisting dilute phase is akin to what we observe for Regime II. Overall, when combined with titrations above and below the overlap concentration, cluster-size distributions can be used as diagnostics of proximity to the critical point. For the measurements to be effective, they will need to be able to access the entirety of the cluster-size distribution. While this is challenging for methods that are based on scattering or fluorescence [118, 123], it should be feasible using methods such as microfluidic resistive pulse sensing (MRPS) that provide a detection range from 70 nm–15 $\mathrm{\mu m}$ [53, 117, 122]. MRPS has been used *in vitro* to quantify the cluster-size distributions in coexisting dilute phases and sub-saturated solutions of different systems [53, 117, 122, 125].

Our calculations show that the percolation line intersects the binodal to the left of the critical point. Thus, the dense phase is a confined physical gel defined by an intra-condensate percolated network [9, 24, 25]. For A1-LCD and related systems, the networks have been shown to have small-world like topology with smaller-scale density inhomogeneities [52]. Quick-freeze deep-etch cryo-electron microscopy of a variant of A1-LCD has provided direct evidence of percolated network-like organization within condensate interiors [117]. Additionally, single-fluorogen imaging of A1-LCD condensates [124] has revealed the inhomogeneous organization of molecules within A1-LCD condensate interiors. These properties give condensates their tunable viscoelasticity, leading to their designation as viscoelastic network fluids [126–131].

In Regime III, the dense phase becomes unconfined and this network swelling has been observed via measurements of volumes and volume fractions by Smokers and Spruijt [47]. Signatures of being in Regime III will be the formation of interconnected networks of coexisting dense and dilute phases. This behavior has been observed for the WNK1 kinase that forms condensates in response to osmotic stress [132]. The observed behavior was referred to as spinodal decomposition. Indeed, Regime III is expected to coincide with the spinodal. Thus, the dynamics of phase separation in Regime III will likely follow that of viscoelastic phase separation [133]. System-spanning networks that are consistent with behaviors in Regime III have also been observed in signaling systems that form at membranes [134].

Our estimation of $T_\mathrm{c}$ was made possible using the formalism of Binder cumulants [64] and rigorous finite-size scaling. Binder cumulants have been used in past to map critical behaviors of different systems. For example, Midya *et al* [135] investigated phase behaviors of semiflexible polymers, focusing on the interplay of nematic ordering, a symmetry-breaking transition, and phase separation. Their work is of direct relevance for investigating the interplay between the formation of amyloid fibrils and phase separation, given that fibrils have been defined using nematic order parameters [136].

Availability of the full binodal allowed us to assess the accuracy of using internal scaling profiles to identify the theta temperature or infer solvent quality. Analysis of internal scaling has been adopted to analyze the molecular form factor in small angle x-ray measurements [17, 137], scaling of efficiencies in single molecule Förster resonance energy transfer measurements [72], and in assessments of solvent quality from simulations of IDPs [138, 139]. Here, we show that this yields an erroneously low estimate for $T_\theta$ and the false result of $T_\mathrm{c}$ being higher than $T_\theta$. Direct computation of $B_2$ provides a more reliable estimate of $T_\theta$ that is in accord with canonical expectations [83]. Calculation of $B_2$ as a function of temperature, as shown here, can be easily achieved because it requires a tiny fraction of the computations that go into obtaining a full binodal.

Our results have broad implications for studies of IDPs. It is customary to use either simulations or measurements to derive internal scaling profiles and quantify the apparent value of $\nu$ [17, 72, 104, 137–141]. The derived $\nu$ value is then used to judge the quality of the solvent for the IDP of interest. Our results suggest that these inferences of solvent quality must be treated with caution. This is because it is quite likely that the temperature or equivalent control parameter at which $\nu = 0.5$ will lie below $T_\mathrm{c}$ or the critical value of the control parameter. Therefore, any rigorous assessment of solvent quality will require direct computation or measurement of the two-body interaction coefficient $B_2$ [40, 142], or alternatively, computation or measurement of phase boundaries [51, 143] as a function of temperature or changes to solvent via chemical perturbations such as changes in pH, denaturant, or salt concentration.

Finally, we note that the findings reported here should also be accessible via off-lattice MD simulations. For each temperature, the simulations will need to be on the order of milliseconds, and the simulation cells will need to encompass $O(10^4)$ molecules. A recent study [144] showed that structures within simulated dense phases of A1-LCD molecules share similarities across three different simulation engines that include LaSSI, MPIPI-recharged [145], and CALVADOS [82, 146]. Based on these comparisons, we propose that the findings reported are likely to be consistent across different simulation protocols that use similar, single-bead-per-residue models. The key to mapping critical points is the avoidance of finite size artifacts and the use of finite size scaling methods [64, 81]. It might also be essential to revisit the use of slab geometries that are in vogue across many MD simulation protocols [20, 57, 59–61, 77, 82, 135, 147]. These geometries are helpful deep inside the mean field regime, but are likely to be problematic in the critical regime. This is because, slabs cause a smoothening of density profiles and this can suppress fluctuations that are the defining hallmarks of critical regions. Assuming finite size artifacts are avoided and cubic boxes that do not break symmetries *a priori* are used, then the extraction of cluster-size distributions across different temperature regimes should be feasible using MD simulations that can be deployed using different engines.

## Data Availability

All data that support the findings of this study are included within the article (and the supporting information). The code for reproducing the results is available at GitHub (https://github.com/Pappulab/phase_behaviors_near_criticality_A1LCD). Supplementary data 1 available at https://doi.org/10.1088/1361-6633/ae70d6/data1.
